# Highly active bacterial DMSP metabolism in the surface microlayer of the eastern China marginal seas

**DOI:** 10.3389/fmicb.2023.1135083

**Published:** 2023-03-23

**Authors:** Xiujie Liu, Yunhui Zhang, Hao Sun, Siyin Tan, Xiao-Hua Zhang

**Affiliations:** ^1^Frontiers Science Center for Deep Ocean Multispheres and Earth System, College of Marine Life Sciences, Ocean University of China, Qingdao, China; ^2^Laboratory for Marine Ecology and Environmental Science, Laoshan Laboratory, Qingdao, China; ^3^Institute of Evolution and Marine Biodiversity, Ocean University of China, Qingdao, China

**Keywords:** DMSP, bacteria, biosynthesis and degradation, sea surface microlayer, subsurface seawater, eastern China marginal seas, spatiotemporal differences

## Abstract

The microbial cycling of dimethylsulfoniopropionate (DMSP) and the resulting gaseous catabolites dimethylsulfide (DMS) or methylmercaptan (MeSH) play key roles in the global sulfur cycle and potentially climate regulation. As the ocean–atmosphere boundary, the sea surface microlayer (SML) is important for the generation and emission of DMS and MeSH. However, understanding of the microbial DMSP metabolism remains limited in the SML. Here, we studied the spatiotemporal differences for DMS/DMSP, bacterial community structure and the key bacterial DMSP metabolic genes between SML and subsurface seawater (SSW) samples in the eastern China marginal seas (the East China Sea and Yellow Sea). In general, DMSP_d_ and DMSP_t_ concentrations, and the abundance of total, free-living and particle-associated bacteria were higher in SML than that in SSW. DMSP synthesis (~7.81-fold for *dsyB*, ~2.93-fold for *mmtN*) and degradation genes (~5.38-fold for *dmdA*, ~6.27-fold for *dddP*) detected in SML were more abundant compared with SSW samples. Free-living bacteria were the main DMSP producers and consumers in eastern Chinese marginal sea. Regionally, the bacterial community structure was distinct between the East China Sea and the Yellow Sea. The abundance of DMSP metabolic genes (*dsyB*, *dmdA*, and *dddP*) and genera in the East China Sea were higher than those of the Yellow Sea. Seasonally, DMSP/DMS level and DMSP metabolic genes and bacteria were more abundant in SML of the East China Sea in summer than in spring. Different from those in spring, *Ruegeria* was the dominant DMSP metabolic bacteria. In conclusion, the DMSP synthesis and degradation showed significant spatiotemporal differences in the SML of the eastern China marginal seas, and were consistently more active in the SML than in the SSW.

## Introduction

Dimethylsulfoniopropionate (DMSP), as one of the most abundant sulfur-containing organic compounds on earth (Kiene et al., 2000), has an estimated annual production of 2.0 Pg (Ksionzek et al., 2016). It is not only an important carbon and sulfur source, but also acts as osmolytes, antioxidants, cryoprotectants and signal molecules in marine organisms (Stefels, 2000; Sunda et al., 2002; Zhang et al., 2019; Zheng et al., 2020). DMSP can be catabolized by bacteria and algae through multiple DMSP lyases, and the resulting dimethyl sulfide (DMS) is the main form of sulfur emission from sea to air (Simo, 2001; Stefels et al., 2007). DMS oxidation products can serve as cloud condensation nuclei, thereby fostering cloud formation and potentially influencing the global climate change (Charlson et al., 1987; Boucher and Pham, 2002; Quinn and Bates, 2011). DMSP can be synthesized by single-cellular phytoplankton (Curson et al., 2018; Kageyama et al., 2018), macroalgae (Challenger and Simpson, 1948; Greene, 1962), angiosperms (Hanson et al., 1994; Kocsis et al., 1998; Otte et al., 2004), corals (Raina et al., 2013) and bacteria (Curson et al., 2017). Three DMSP synthesis pathways have been identified with methionine (Met) as the starting substrate: methylation pathways (angiosperms, bacteria; Williams et al., 2019), transamination pathway (marine algae, corals, bacteria; Gage et al., 1997; Curson et al., 2017), and decarboxylation pathway (dinoflagellates; Uchida et al., 1996).

Recent studies have identified the key S-methyltransferase encoding genes of the Met transamination (*dsyB*) and Met methylation (*mmtN*) pathways in marine bacteria (Curson et al., 2017; Williams et al., 2019). It is estimated that ~0.35% of marine bacteria (mainly *Alphaproteobacteria*) contain *dsyB* (Curson et al., 2018), which was far more abundant than the *mmtN* gene (mainly in *Alphaproteobacteria*, *Gammaproteobacteria* and *Actinobacteria*; Williams et al., 2019; Sun et al., 2020; Zheng et al., 2020). The *dsyB* and *mmtN* genes have been identified as key genes for bacterial DMSP production, and were often used to predict the ability of bacterial DMSP synthesis in the environment (Curson et al., 2017; Williams et al., 2019). Eukaryotic DMSP producing enzymes, DSYB and TpMMT, are also the key reporters for generating DMSP *via* Met transamination and methylation (Curson et al., 2018; Kageyama et al., 2018). There are many bacterial genera that produce DMSP but lack *dsyB* or *mmtN* in their genomes and likely have isoform enzymes or novel pathways, such as *Marinobacter* (Curson et al., 2017; Williams et al., 2019). Phytoplankton, such as dinoflagellates, diatom and green algae are considered to be the main oceanic DMSP producers (Zhang et al., 2019). However, bacteria also significantly contribute to marine DMSP production, especially in aphotic and deep seawater and surface marine sediments where phytoplankton are scarce (Williams et al., 2019; Song et al., 2020; Sun et al., 2020; Zheng et al., 2020; Liu et al., 2021; Zhang et al., 2021).

Marine bacteria are considered as primary contributors to DMSP catabolism although many marine phytoplankton can also catabolize DMSP (Zubkov et al., 2001; Alcolombri et al., 2015; Curson et al., 2017). There are three known DMSP catabolic pathways: demethylation pathway (Curson et al., 2011), cleavage pathway (Curson et al., 2011), and oxidation pathway (Thume et al., 2018). Most DMSP (~75%) is catabolized through demethylation pathway generating the active gas methanthiol (MeSH; Howard et al., 2006). Gene encoding the key enzyme in demethylation pathway, *dmdA*, can be divided into five clades (clade A, B, C, D, E) and 14 subclades, of which C/2 and D/1 are the most abundant subclades in the oceans (Cui et al., 2015; Liu et al., 2018). DmdA are widely distributed in marine bacteria such as *Roseobacter*, SAR11 clade, SAR116 clade and *Gammaproteobacteria*, as well as in bacteriophages on coral edges (Howard et al., 2008; Raina et al., 2010). The cleavage pathways account for ~10% of DMSP catabolism and are mediated by DMSP lyases (Kiene et al., 2000). Nine DMSP lyases have yet been discovered in bacteria (*dddD*, *dddL*, *dddP*, *dddQ*, *dddW*, *dddY*, *dddK*, and *dddX*; Curson et al., 2011; Sun et al., 2016; Zhang et al., 2019; Li et al., 2021) and algae (*Alma1*; Alcolombri et al., 2015). *dddP* is the most abundant *ddd* genes in the marine environments (~8%; Curson et al., 2018), and is widely used as a key reporter for environmental DMSP cleavage (Liu et al., 2018). DddP are predominantly in *Roseobacter*, SAR11 clade, SAR116 clade and some *Gammaproteobacteria* (Curson et al., 2008, 2011).

The sea surface microlayer (SML), the uppermost 1–1,000 μm of sea surface water, is an active interface for material exchange and global biogeochemical cycling between atmosphere and seawater (Hardy, 1982; Cunliffe and Murrell, 2009; Wurl et al., 2011). Compared with the subsurface seawater (SSW), the SML is generally more physically stable, more environmentally stressed, and enriched with both particulate matter and organic compounds (Yang et al., 2005b). The SML can be enriched up to 10^2^–10^3^ times in molecular and dissolved organic matters, and its composition may vary horizontally, seasonally or even from day to night (Perliński et al., 2017). SML is also generally rich in bacteria and microalgae, which can be regarded as a unique interface separating two ecosystems (Liss and Duce, 1997). Previous studies indicated that bacteria were more abundant in SML than in SSW in different regions (Sun et al., 2020). Moreover, higher DMSP and DMS levels in the SML have been reported in different areas and seasons when compared with those in the SSW (Yang et al., 2005a,b, 2008; Zhang et al., 2008; Sun et al., 2020). Our previous work showed the abundance of DMSP metabolic bacteria and functional genes was higher in SML than in SSW of the East China Sea (ECS) during spring (Sun et al., 2020). However, whether abundant DMSP metabolic bacteria presented in the SML in different regions and seasons were yet to be investigated.

The East China Sea (ECS) and the Yellow Sea (YS) are China’s marginal seas with high primary productivity due to the influence of warm currents and terrestrial inputs (Lee and Chao, 2003; Lin et al., 2005; Yeh et al., 2015). In this study, DMSP/DMS levels and the abundance of DMSP metabolic bacteria and genes (in the free-living and particle-associated factions) were investigated to compare bacterial DMSP metabolism in the SML and the SSW of the eastern China marginal seas. Differences of DMSP metabolism between seasons (spring and summer) in SML of the ECS were also discussed based on our previous study (Sun et al., 2020). These results emphasize the important roles of marine SML bacteria in DMSP metabolism.

## Materials and methods

### Sampling and environmental parameters

**Figure 1:**
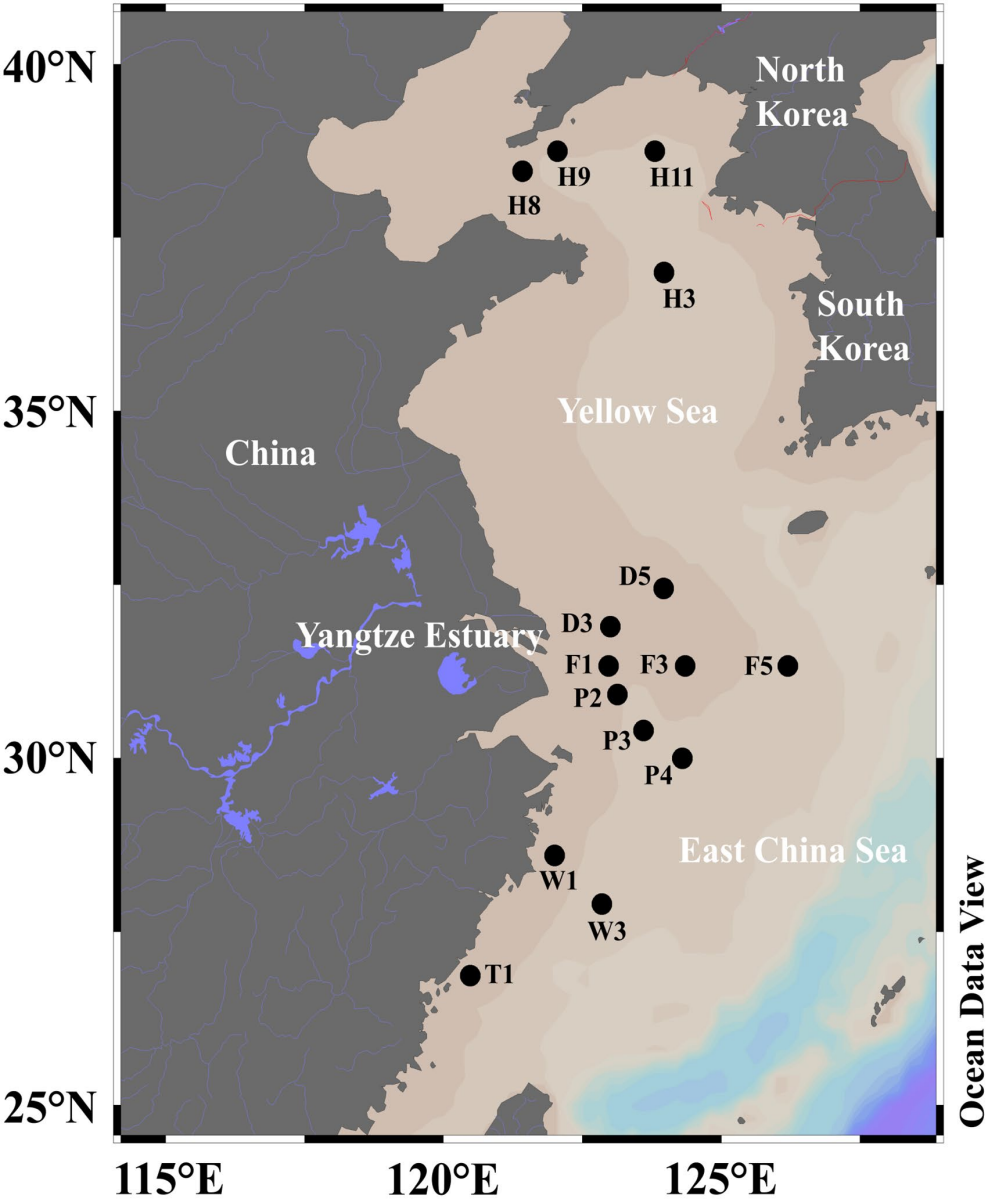
The sampling sites of SML and SSW in the eastern China marginal seas in summer.

SML (0–1 mm depth) and SSW (2.5–5.0 m depth) waters were collected from 15 sites of the ECS (D3, D5, F1, F3, F5, P2, P3, P4, W1, W3, T1) and the YS (H8, H9, H11, H3) aboard the R/V *Dongfang Hong* 2 in June 2018 (Figure 1; Supplementary Table 1). SML water samples were collected by using the Garrett metal screen (MS; Agogué et al., 2004; Chen et al., 2016), whereas SSW samples were collected with a Sealogger CTD (SBE25, Electronic Inc., United States) rosette water sampler (~4 m below the surface). Samples containing 1 L seawater were filtered serially through 3 and 0.22 μm polycarbonate membranes (Millipore Corporation, Billerica, MA, United States), respectively. The particle-associated (PA) bacteria in the seawater were collected through 3 μm polycarbonate membranes, and the free-living (FL) bacteria in the seawater were collected through 0.22 μm polycarbonate membranes. For the quantification of *Synechococcus* (SYN), *Prochlorococcus*, picoeukaryotes (PEUK) and heterotrophic bacteria, 2 ml of water samples from each sample were placed into sterile tubes and immediately fixed with paraformaldehyde (final concentration 4%, v/v) for 30 min in the dark at room temperature. Liquid nitrogen was immediately used to freeze both membranes and 2 ml water samples, which were then stored at −20°C on board and transferred to −80°C in the laboratory.

Hydrological parameters (temperature, salinity and depth) were obtained by CTD equipped on the water sampler *in situ*. The concentrations of Chlorophyll *a* (Chl *a*) were measured as described previously (Zhang et al., 2014). Using GF/F filter (Whatman) with a pore size of 0.7 μm immediately filtered seawater samples after collection on board, which were then soaked in 90% (v/v) acetone in the dark for 24 h to extract Chl *a*. The F4500 fluorescence spectrophotometer was used to determine the concentration of Chl *a* in the extracts (Hitachi, Japan). DO was measured by Winkler method (Carpenter, 1965). Samples for nutrients (PO_4_^3−^, NO_2_^−^, NO_3_^−^, SiO_3_^2−^, and NH_4_^+^) were filtered with 0.45 μm cellulose acetate membranes and were analyzed by an Auto-Analyzer (AA3, Seal Analytical Ltd., United Kingdom; Liu et al., 2015). The abundances of *Synechococcus*, *Prochlorococcus*, picoeukaryotes, and heterotrophic bacteria were measured by flow cytometer (BD FACSJazz, United States) in the laboratory (Zhao et al., 2017).

### The DMS and DMSP concentration measurement

DMS and DMSP concentrations in seawater samples were measured *in situ* as described previously (Yang et al., 2011). DMS in seawater samples was captured by a cryogenic purge-and-trap pretreatment system and the concentration of DMS was measured by flame photometric detection with gas chromatography (Agilent GC-7890B; Tan et al., 2017). To avoid the influence of microorganism cell rupture caused by filtration pressure on DMSP concentrations, DMSP was captured by gravity filtration method (Tan et al., 2017). Total DMSP (DMSP_t_) refers to the DMSP without filtration; Particulate DMSP (DMSP_p_) refers to the DMSP that captured on the 0.45 μm filter membrane; Dissolved DMSP (DMSP_d_) refers to the DMSP in the filtrate through the 0.45 μm filtration membrane.

### Total DNA extraction

Total DNA were extracted from 3 and 0.22 μm membranes of the SML and SSW samples using the Phenol-chloroform method (Sun et al., 2020). The extracted DNA was redissolved in 10 mM Tris–HCl (pH 8.0) and stored at −80°C. Subsequently, it was used for bacterial 16S rRNA high-throughput sequencing and qPCR to quantify functional genes.

### Quantitative PCR

The abundances of total bacterial 16S rRNA genes, DMSP producing genes *dsyB*, *mmtN*, catabolic genes *dmdA* (C/2, D/1 subclade) and *dddP* in seawater samples were quantified by qPCR. All primer sequences and annealing temperatures were listed in Table 1. PCR reactions and melting curves were performed as described by Sun et al. (2020). qPCR standard curve was made using pUCm-T vector (Biotech, China) that contained a single copy of the corresponding gene. Plasmids were extracted with Mini Plasmid Kit (Takara, Tokyo, Japan), linearized with restriction endonuclease XhoI, purified with TIANgel Mini Purification Kit (TIANGEN Biotech, Beijing) and quantified with Nanodrop-1,000 spectrophotometer. A standard curve was then generated using 10-fold serially diluted linearized plasmids, all of which showed linear correlations of R2 = 0.99. The qPCR gene amplification efficiency ranged from 95 to 105% (93% to 98% for *dsyB* and *mmtN*). Three techniques replicates were set for each sample. Double-distilled water was used as a template for negative control. The qPCR of all samples was performed on the StepOne ™ Real-time PCR System (Applied Biosystems), and the obtained data were analyzed by the StepOne software (version 2.2). The abundance of each gene in the sample was calculated according to the copy number obtained by qPCR and the volume of filtered water sample.

**Table 1:** Primers and amplification conditions for qPCR detection and high-through sequencing of bacteria.

Target gene	Primers	Sequences (5′-3′)	Amplicon length (bp)	Annealing temp (°C)	Usage	References
16S rRNA	338F	ACTCCTACGGGAGGCAGCAG	180	53	qPCR	Yin et al. (2013)
	518R	ATTACCGCGGCTGCTGG				
*dsyB*	dsyBF	CATGGGSTCSAAGGCSCTKTT	246	61		Williams et al. (2019)
	dsyBR	GCAGRTARTCGCCGAAATCGTA				Williams et al. (2019)
*mmtN*	mmtNF	CCGAGGTGGTCATGAAYTTYGG	301	54		Williams et al. (2019)
	mmtNR	GGATCACGCACACYTCRTGRTA				Williams et al. (2019)
*dddP*	874F	AAYGAAATWGTTGCCTTTGA	97	41		Levine et al. (2012)
	971R	GCATDGCRTAAATCATATC				
*dmdA*(C/2)	291F	AGATGAAAATGCTGGAATGATAAATG	191	50		Levine et al. (2012)
	482R	AAATCTTCAGACTTTGGACCTTG				Varaljay et al. (2010)
*dmdA*(D/1)	268F	AGATGTTATTATTGTCCAATAATTGATG	89	49		Levine et al. (2012)
	356R	ATCCACCATCTATCTTCAGCTA				Varaljay et al. (2010)
16S rRNA	515modF	GTGYCAGCMGCCGCGGTAA	291	50	Amplicon sequencing	Walters et al. (2016)
	806modR	GGACTACNVGGGTWTCTAAT				

### Bacterial 16S rRNA gene amplicon sequencing and analysis

The total bacterial 16S rRNA gene was amplified by Majorbio Bio-Pharm Technology Co. Ltd. (Shanghai, China) using primers 515modF and 806modR (Walters et al., 2016). The PCR amplification system (20 μl) was conducted as follows: 4 μL of 5 × Fast Pfu buffer, 2 μL of 2.5 mM dNTPs, 0.8 μL of 5 μM forward and reverse primers, 1 U of TransStart Fastpfu DNA polymerase, 10 ng of template DNA, 0.2 μL of BSA (bovine serum albumin), and add double-distilled water to 20 μL. The PCR cycling condition was conducted as follows: a. pre-denaturation at 95°C for 3 min, b. 29 cycles of denaturation at 95°C for 30 s, annealing at 55°C for 30 s, extension at 72°C for 45 s, c. extension at 72°C for 10 min, and 10°C until halted. The PCR amplification product was purified using the AxyPrep DNA Gel Extraction Kit (Axygen Biosciences, Union City, CA, United States), and the DNA was quantified using QuantiFluor™-ST (Promega, United States). The purified amplicons were merged in equimolar and paired-end sequenced on the Illumina MiSeq platform (Illumina, San Diego, United States) according to the standard protocols of Majorbio Bio-Pharm Technology Co. Ltd. (Shanghai, China). After subsampling each sample to an equal sequencing depth according to the minimum number (65,552) of sample sequences, OTUs were clustered using Usearch7.0 method of the QIIME1.9.1 with 97% similarity cutoff. The taxonomic position of each OTU representative 16S rRNA gene sequence was analyzed by Silva 128 16S rRNA database^1^ using confidence threshold of 70%. The absolute abundance of potential DMSP biosynthetic and catabolic genera were estimated by their relative abundance determined by 16S rRNA gene amplicon sequencing and the total bacteria abundance quantified by qPCR analysis of 16S rRNA gene. This is a semi-quantitative approach as it is solely based on the presence of these genes in isolates/genomes belonging to similar genera reported in previous publications.

### Statistical analysis

Mothur^2^ was used to calculate the Alpha diversity indices such as Shannon, Chao1 and Good’s coverage to measure the species richness and diversity of the community (Sun et al., 2016; Bullock et al., 2017). For beta diversity, non-metric multidimensional scaling analysis (NMDS) and hierarchical clustering trees were performed with ANOSIM based on Bray-Curtis distance matrices using the “vegan” package in R software (version 4.1.1). The difference of bacterial community structure between the SML and SSW was analyzed by Wilcoxon rank-sum test. The differences in bacterial diversity and richness between SML and SSW were analyzed by Wilcoxon signed-rank test. The relationship between environmental factors and bacterial community structure was evaluated by distance-based redundancy analysis (db-RDA) with 999 Monte Carlo permutation tests using the Canoco software (version 5.0, Microcomputer Power). The correlations between environmental factors and functional gene abundance were conducted using Spearman correlation test. The difference of environmental factors and functional gene abundance between SML and SSW was conducted by Wilcoxon signed-rank test. Differences in environmental factors and gene abundance between seasons and between regions were conducted using the Mann–Whitney tests. All statistical analyses were performed on SPSS version 25.0 (SPSS, Chicago, IL, United States) and the significance threshold for all tests was set with *p* < 0.05 and *p* < 0.01. The map of sampling sites was created using Ocean Data View (ODV, v5.1.7) and figures were drawn by Origin 2021 software^3^ or GraphPad Prism 6.01.

### Data availability

Raw reads from the summer 16S rRNA gene amplicon sequencing have been deposited in the NCBI BioProject database under the accession number PRJNA648032. Raw reads from the spring 16S rRNA gene amplicon sequencing were deposited into the NCBI Sequence Read Archive (SRA) database with accession number SRP174872 under the BioProject PRJNA511511 (Sun et al., 2020).

## Results

### DMSP concentrations and other environmental parameters

The environmental parameters of all samples were listed in Supplementary Table 1. In the eastern China marginal seas (ECS and YS), DOC was significantly higher in the SML than in the SSW (*p* < 0.05, Wilcoxon signed-rank tests, Supplementary Tables 2–4). DMSP_d_ and DMSP_t_ concentrations were significantly higher in the SML (162.66 ± 324.28 nM and 403.09 ± 647.46 nM) than in the SSW samples (7.51 ± 3.94 nM and 91.49 ± 55.22 nM; ~21.67-fold and 4.41-fold, respectively, *p* = 0.001 and 0.019, Wilcoxon signed-rank tests, Supplementary Table 3). DMS, DMSP_p_ and Chl *a* concentrations showed no significant difference between the SML and SSW samples (Supplementary Tables 2–4).

Regionally, DOC in the ECS (130.34 ± 30.65 μmol C/L) was lower than that of the YS (195.75 ± 26.97 μmol C/L), while Chl *a* of the ECS (2.01 ± 1.21 μg/L) was higher than that of the YS (0.50 ± 0.18 μg/L) in the SML (Supplementary Table 1). The DMS and DMSP concentrations reached the maximum at the SML site F3 of the ESC among all samples (26.67 nM and 2123.95 nM, respectively, Supplementary Figure 1). DMS and DMSP_t_ concentrations of the SML in the ECS were higher (~1.61-fold and ~2.47-fold) than in the YS (Supplementary Figure 1; Supplementary Table 1), while no difference was observed for DMS and DMSP_t_ concentrations in the SSW of the ECS and YS (*p* > 0.05, Supplementary Figure 1; Supplementary Table 5).

In both spring and summer, DMSP_d_ and DMSP_t_ concentrations were higher in SML than in SSW, and DMS and Chl *a* were not significantly different between these two water layers (Supplementary Figure 1; Supplementary Tables 1, 3, 4; Sun et al., 2020). Additionally, the concentrations of DMS, DMSP_d_, DMSP_p_ and DMSP_t_ in the summer SML samples were higher than those in spring (Supplementary Table 5). Chl *a* in the SML of the ECS in summer samples was also higher (~2.00-fold) than that in spring (Sun et al., 2020).

### The abundance of bacteria and eukaryotes

The total abundance of the bacterial 16S rRNA gene (the sum of FL and PA bacteria, Supplementary Figure 2B) quantified by qPCR was consistent with the changes of heterotrophic bacteria number (Supplementary Figure 2C) shown by flow cytometer in SML and SSW of the eastern China marginal seas. The average counting of heterotrophic bacteria in the SML (1.72 ± 3.02 × 10^9^ cells L^−1^) was higher than in the SSW (7.57 ± 6.30 × 10^8^ cells L^−1^). Total abundance of the bacterial 16S rRNA gene in SML (2.91 ± 3.28 × 10^9^ copies L^−1^) was also significantly higher than in SSW (6.36 ± 4.95 × 10^8^ copies L^−1^，*p* < 0.01, Wilcoxon signed-rank tests, Supplementary Figure 2B; Supplementary Table 2), and was more abundant in FL fraction than in the PA fraction (~8.28-fold for SML and ~ 7.27-fold for SSW, respectively, Supplementary Figure 2A).

Regionally, the average counting of heterotrophic bacteria in the ECS SML (2.10 ± 3.53 × 10^9^ cells L^−1^) was higher than those of the YS SML (7.56 ± 5.14 × 10^8^ cells L^−1^), so was that for the SSW samples (9.03 ± 7.16 × 10^8^ cells L^−1^ in the ECS and 3.58 ± 1.83× 10^8^ cells L^−1^ in the YS). Total abundance of the bacterial 16S rRNA gene in the ECS (3.30 ± 3.71 × 10^9^ copies L^−1^ in the SML and 7.90 ± 4.93 × 10^8^ copies L^−1^ in the SSW) was also higher than those of the YS SML (1.81 ± 1.52× 10^9^ copies L^−1^ in the SML and 2.11 ± 0.88 × 10^8^ copies L^−1^ in the SSW; Supplementary Figure 2B; Supplementary Tables 3, 4). The abundance of both FL and PA bacteria was significantly higher in the SML of the ECS than in the SSW (*p* < 0.05, Wilcoxon signed-rank tests, Supplementary Table 3), but there was no significant difference between the two water layers in the YS (*p* > 0.05, Wilcoxon signed-rank tests, Supplementary Table 4). Seasonally, the total abundance of bacterial 16S rRNA gene in summer SML and SSW samples was ~3.00-fold and ~ 5.42-fold higher than those in spring (1.10 ± 0.57 × 10^9^ copies L^−1^ for the SML and 1.46 ± 0.91 × 10^8^ copies L^−1^ for the SSW, Sun et al., 2020), respectively.

For eukaryotes in the eastern China marginal seas, picoeukaryotes and *Synechococcus* were more abundant in the SSW (7.74 ± 17.65 × 10^5^ and 1.31 ± 1.84 × 10^7^ cells L^−1^) than in the SML (1.17 ± 2.55 × 10^5^ and 1.06 ± 1.46 × 10^7^ cells L^−1^, Supplementary Table 1). *Synechococcus* (SYN) in the ECS SML (1.71 ± 1.89 × 10^7^ cells L^−1^) was more abundant than that in the YS SML (1.72 ± 1.03 × 10^6^ cells L^−1^; *p* < 0.05, Mann–Whitney tests, Supplementary Table 4).

### α- and β-diversity of eastern China marginal seas samples

**Figure 2:**
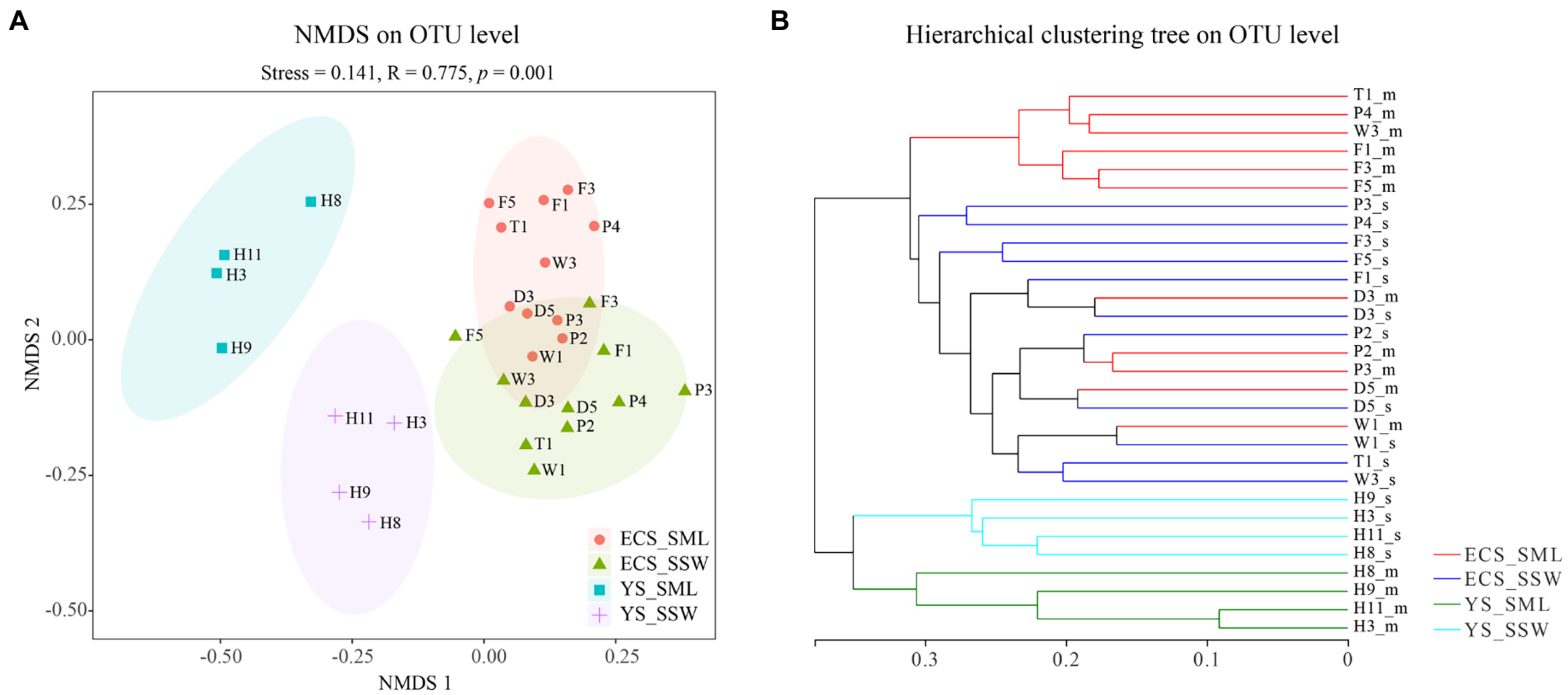
The NMDS analysis and hierarchical clustering tree of bacteria community of the SML and SSW in the eastern China marginal seas in summer. **(A)**, The NMDS analysis on OTU level; **(B)**, The hierarchical clustering tree on OTU level. ECS_SML, the East China Sea SML samples; ECS_SSW, the East China Sea SSW samples; YS_SML, the Yellow Sea SML samples; YS_SSW, the Yellow Sea SSW samples.

In total, 2,871,910 reads were obtained with an average sequence length of 273 bp *via* the 16S rRNA gene amplicon sequencing. After quality control and subsampling, a total of 3,219 OTUs were assigned at the 97% sequence similarity threshold level. The good’s coverage values (99.58%–99.76%, Supplementary Table 6) indicated that the sequencing results can cover most of the bacterial community in the samples. The NMDS analysis and hierarchical clustering trees based on Bray-Curtis distances showed a clear separation of communities by sampling site. Basically, all the samples were partitioned into four geographic clusters (stress = 0.141), i.e., ECS_SML, ECS_SSW, YS_SML, and YS_SSW (Figure 2). The Shannon and Chao 1 indices were used as indicators of the bacterial community diversity and richness in SML and SSW samples, respectively (Supplementary Table 6). Differences of the bacterial community of SML and SSW in the whole eastern China marginal seas, and differences of the bacterial community of SML and SSW between ECS and YS and between summer and spring were analyzed (Supplementary Figures 3–6). In general, the Shannon diversity index was significantly higher in the SSW than in the SML (*p* < 0.05, Wilcoxon signed-rank test, Supplementary Figure 4A), while the Chao 1 index was not significant different between the SML and SSW samples (*p* > 0.05, Wilcoxon signed-rank test, Supplementary Figure 4B). Regionally, the Chao 1 and Shannon diversity indices in the ECS SML were significantly higher than that of YS SML (*p* < 0.05, Wilcoxon signed-rank test, Supplementary Figures 5A,B). The bacterial diversity in the YS SSW was higher than that of SML (*p* < 0.01, Wilcoxon signed-rank test, Supplementary Figure 5A). Both the Chao 1 and Shannon diversity indices showed no significant differences between SML and SSW samples of the ECS in summer, which were significantly higher than those in spring (*p* < 0.001, Wilcoxon signed-rank test, Supplementary Figures 5C,D).

### Bacterial community and influence of environmental factors

**Figure 3:**
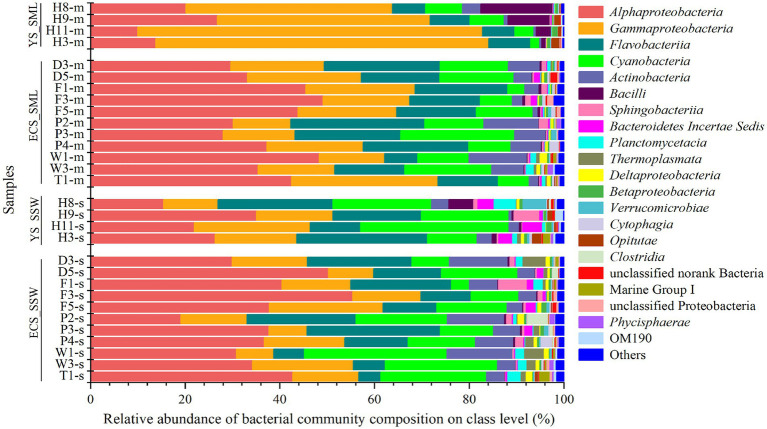
The bacteria community of SML and SSW in the eastern China marginal seas in summer. The SML and SSW samples are indicated by “m” or “s” in their sample names, respectively. YS_SML, the Yellow Sea SML samples; ECS_SML, the East China Sea SML samples; YS_SSW, the Yellow Sea SSW samples; ECS_SSW, the East China Sea SSW samples.

Microbial community of SML and SSW samples from the eastern China marginal seas was analyzed to identify potential DMSP producers and consumers *via* 16S rRNA gene amplicon sequencing. From the eastern Chinese marginal sea, it possesses more *Gammaproteobacteria* in the SML, while *Cyanobacteria*, Bacteroidetes Incertae Sedis, *Thermoplasmata*, *Verrucomicrobiae*, and *Clostridia* were significantly more abundant in the SSW (*p* < 0.05; Supplementary Figure 3A). The relative abundance of *Alphaproteobacteria* was not significantly different between the two layers (Figure 3). At the genus level, the abundance of *Pseudoalteromonas*, *Erythrobacter*, *Psychrobacter*, *Vibrio*, *Halomonas*, *Pseudomonas* were higher in the SML (*p* < 0.05; Supplementary Figure 3B). Meanwhile, the structure of bacterial communities in the ECS and YS was significantly distinct (Figure 3).

Bacterial communities in SML and SSW of the ECS were both dominated by *Alphaproteobacteria*, followed by *Gammaproteobacteria*, *Flavobacteria*, *Cyanobacteria* and *Actinobacteria* (Figure 3; Supplementary Figure 6A). *Synechococcus* and *Erythrobacter* were the most abundant genera in both SML and SSW samples, followed by unclassified Surface 1, NS5 marine group, *Candidatus* Actinomarina, *Ruegeria*, *Paracoccus*, norank SAR86 clade, *Alteromonas* and *Sulfitobacter*. Among them, *Erythrobacter* and *Psychrobacter* were more abundant in the SML (*p* < 0.001, Wilcoxon rank-sum test), while norank SAR86 clade was more abundant in the SSW (*p* < 0.05, Wilcoxon rank-sum test, Supplementary Figure 6B). Unlike in summer, the bacterial communities of SML and SSW in spring were dominated by *Gammaproteobacteria*, followed by *Alphaproteobacteria*, *Actinobacteria* and *Flavobacteria* (Sun et al., 2020).

Bacterial community structure in the YS was dominated by *Gammaproteobacteria* (43.63%–72.80% in SML and 11.54%–24.52% in SSW, respectively), which was significantly higher in SML than that of SSW (*p* < 0.05, Wilcoxon rank-sum test, Figure 3; Supplementary Figure 6C). *Pseudoalteromonas* (14.03%–52.00% in SML and 0.57%–11.05% in SSW, respectively) was the most abundant genera in the YS. Additionally, *Alphaproteobacteria*, *Flavobacteria* and *Cyanobacteria* were also with higher proportions in SML than in SSW samples. At the genus level, the relative abundances of *Acinetobacter*, *Alteromonas*, *Formosa*, *Halomonas*, *Pseudomonas* and *Vibrio* were higher in SML than those in SSW (*p* < 0.05, Wilcoxon rank-sum test, Supplementary Figure 6D).

**Figure 4:**
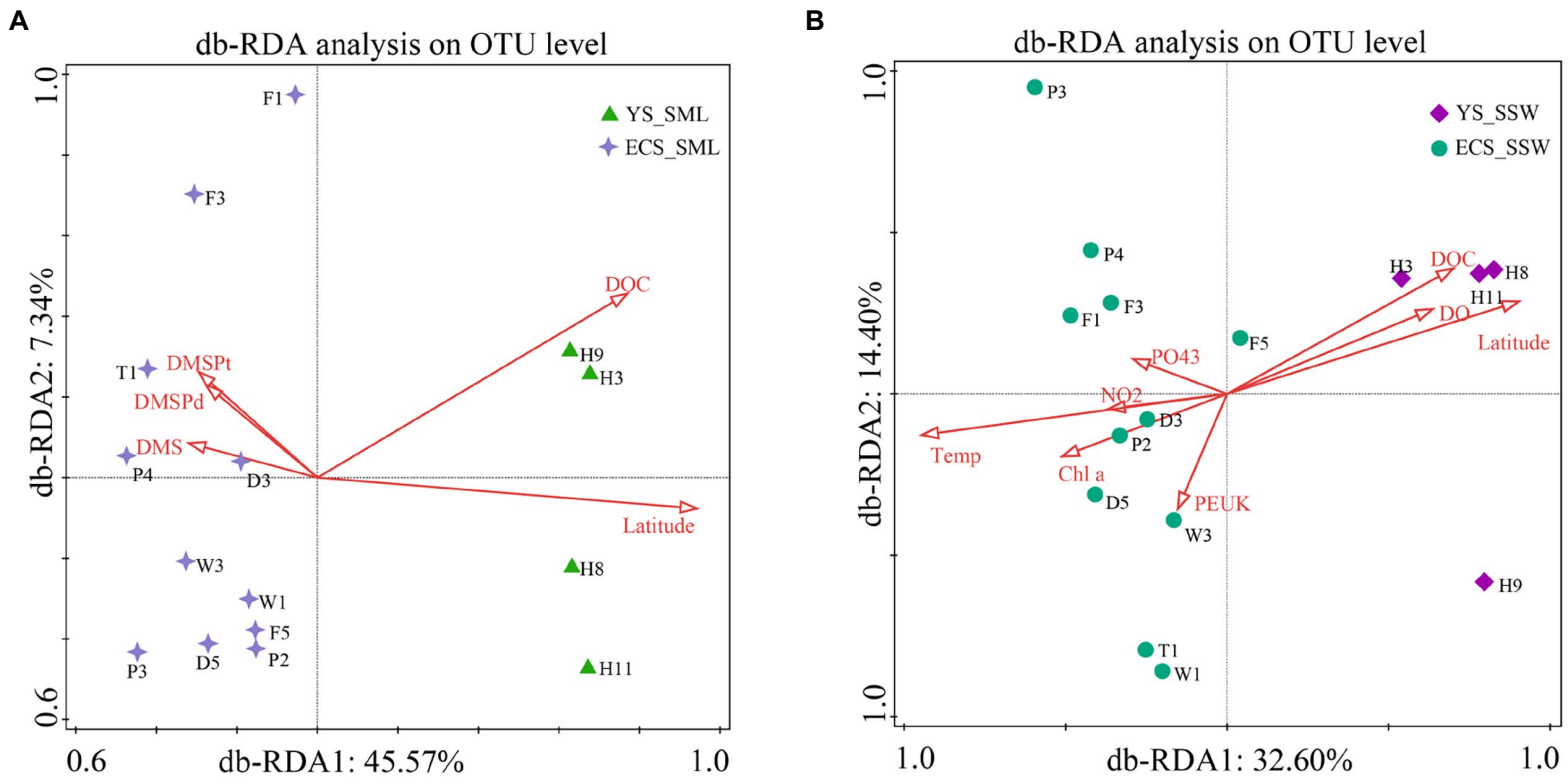
db-RDA analysis showing the relationship between bacterial community and environmental factors of the SML and SSW in the East China Sea and the Yellow Sea in summer. **(A)**, db-RDA analysis of the SML samples; **(B)**, db-RDA analysis of the SSW samples. YS_SML, the Yellow Sea SML samples; ECS_SML, the East China Sea SML samples; YS_SSW, the Yellow Sea SSW samples; ECS_SSW, the East China Sea SSW samples.

To understand the influence of environmental factors on microbial communities, the predictor variables of environmental factors in SML and SSW of the ECS and YS were analyzed by db-RDA based on Bray-Curtis distances (Figure 4). Latitude, DOC, DMS, DMSP_d_ and DMSP_t_ were significantly correlated with the distribution of bacterial communities in SML samples (explained 45.57% by first axis and 7.34% by second axis, Figure 4A). Environmental factors (latitude, DOC, DO, Chl *a*, temperature, PEUK, PO_4_^3−^ and NO_2_^−^) were the main contributors affecting the bacterial community structure of SSW in the ECS and YS (explained 32.60% by first axis and 14.40% by second axis, Figure 4B). Different from the main influencing factors (longitude and DMS) in spring (Sun et al., 2020), the bacterial community of the ECS in summer was mainly affected by latitude, DOC, temperature, DMSP, PEUK and Chl *a*.

### Variation of DMSP biosynthesis and catabolic gene abundance in SML and SSW

**Figure 5:**
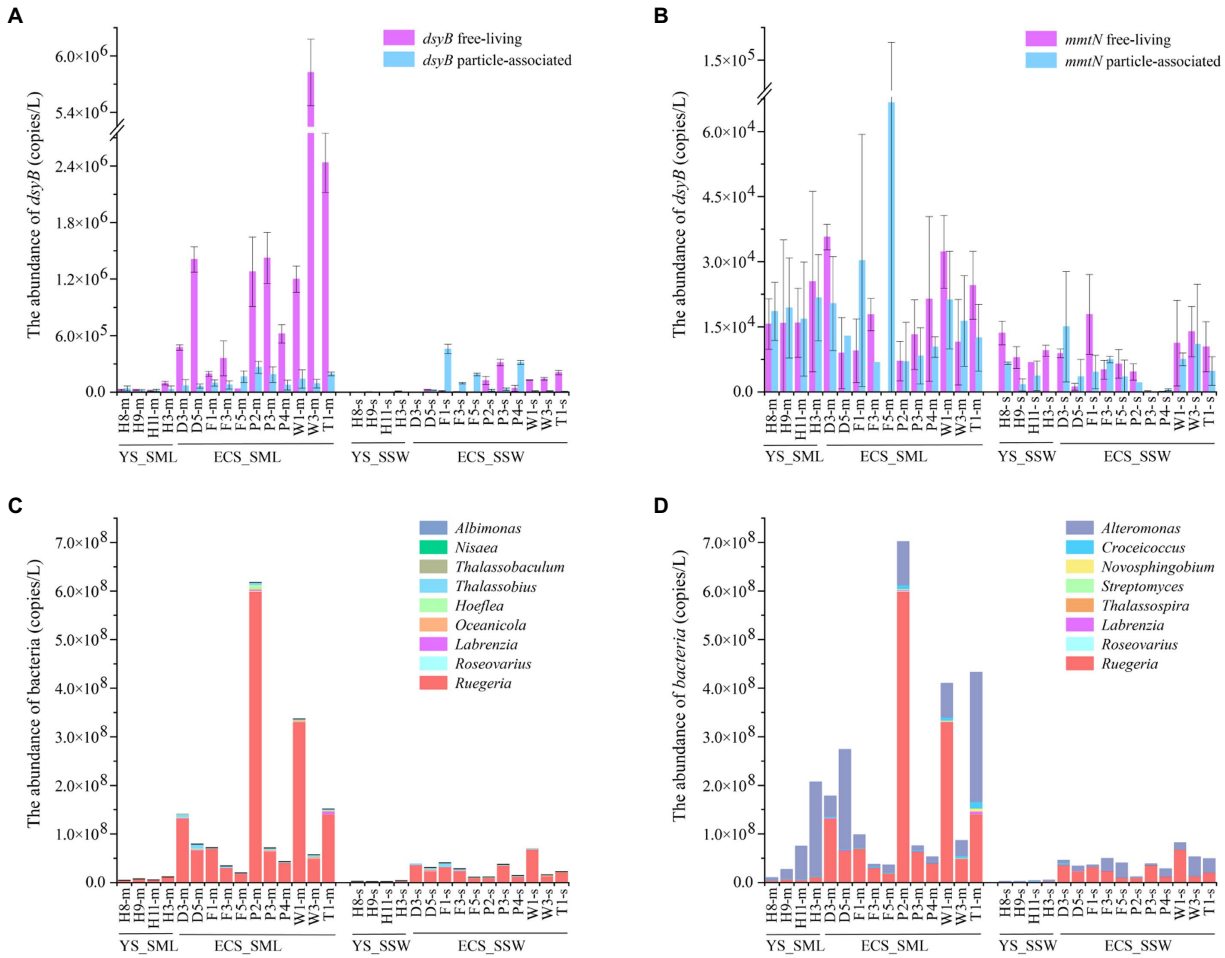
The abundance of DMSP-producing organisms and genes in the SML and SSW samples from the East China Sea and the Yellow Sea in summer. **(A)**, The abundance of *dsyB* determined by qPCR. **(B)**, The abundance of *mmtN* determined by qPCR. **(C)**, The abundance of genera with representatives known to contain *dsyB*. **(D)**, The abundance of genera with representatives known to contain *mmtN*. Three technical replicates are set for each sample. The SML and SSW samples are indicated with “m” or “s” in their sample names, respectively. YS_SML, the Yellow Sea SML samples; ECS_SML, the East China Sea SML samples; YS_SSW, the Yellow Sea SSW samples; ECS_SSW, the East China Sea SSW samples.

As the dominant DMSP biosynthesis gene in the eastern Chinese marginal sea, the total abundance of *dsyB* and *mmtN* in the SML were ~7.81 and 2.93 folds higher than in the SSW (*p* < 0.01, Wilcoxon signed-rank tests, Figure 5A; Supplementary Figure 7A; Supplementary Table 2). *dsyB* was generally more abundant (~33.79-fold) than *mmtN* (Supplementary Figures 7A,B) in all SML and SSW samples. *dsyB* also showed higher abundance in the FL fractions (1.03 ± 1.51 × 10^6^ copies L^−1^) than in the PA fractions (1.01 ± 0.73 × 10^5^ copies L^−1^) in the SML (Figure 5A), whereas the *mmtN* did not differ between these two lifestyles (Figure 5B). The abundance of *dsyB* showed an increasing trend from north to south, and was negatively correlated with latitude (*p* < 0.01, Spearman correlation tests, Supplementary Table 7). Moreover, we found that *dsyB* was negatively correlated with DOC in the SML, and significantly correlated with DO and temperature in the SSW (*p* < 0.05, Wilcoxon signed-rank tests, Supplementary Tables 7, 8). *mmtN* (especially the FL fraction) was correlated with longitude, pH, and DMSP in the SSW, while it was not found to be influenced by environmental factors in the SML (*p* < 0.01, Spearman correlation tests, Supplementary Tables 7, 8).

Geographically, *dsyB* gene abundance in the ECS was higher than that of the YS (~21.87-fold in SML, ~29.31-fold in SSW, respectively), but no significant difference was found for *mmtN* (Supplementary Figures 7A,B; Supplementary Table 5). Compared with the ECS samples in spring, *dsyB* and *mmtN* of the SML and SSW samples were significantly more abundant in summer (*p* < 0.01, Mann–Whitney tests, Supplementary Table 5), and the abundance of DMSP biosynthesis genes were consistently higher in the SML than in the SSW among both summer and spring samples.

**Figure 6:**
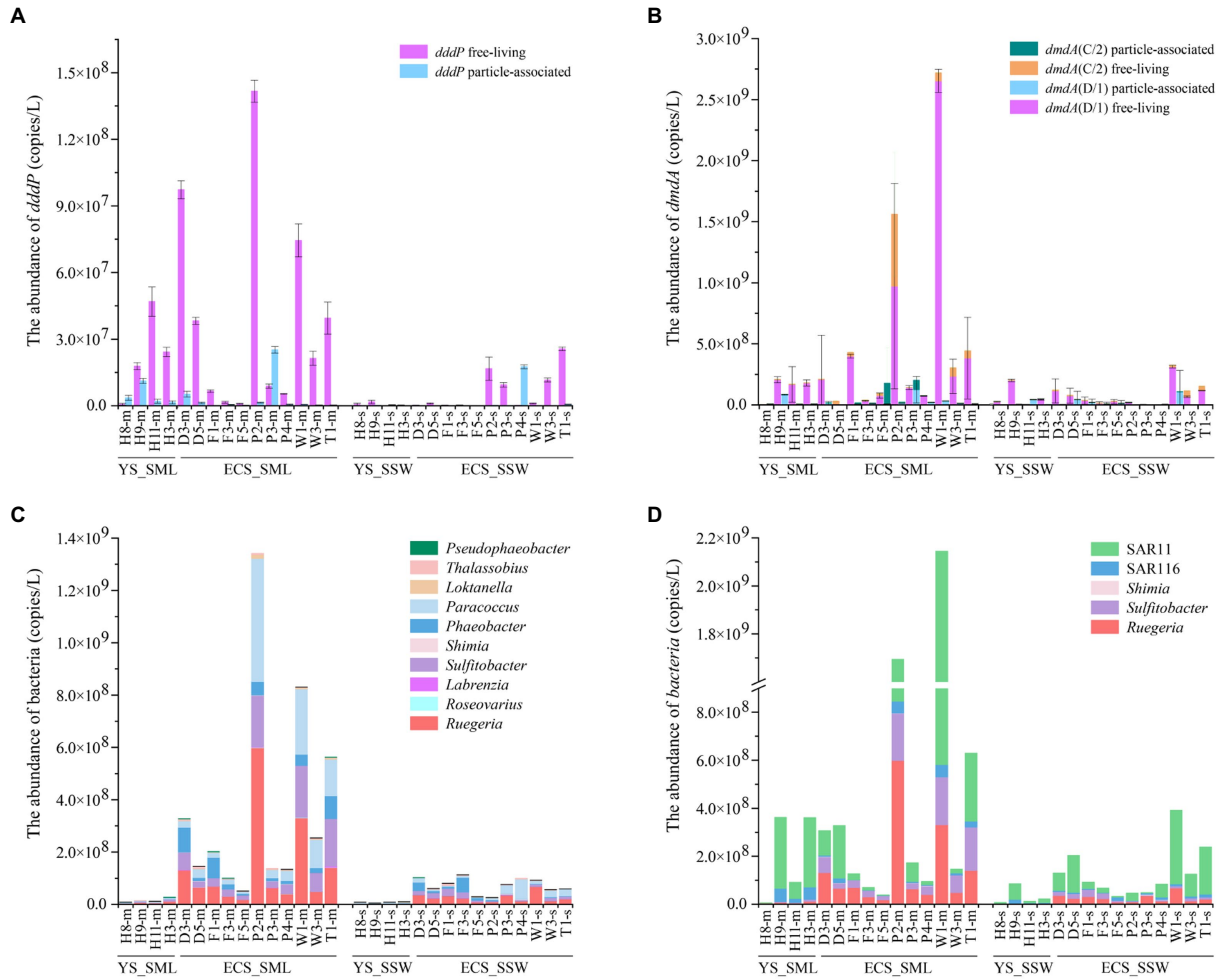
The abundance of DMSP catabolic organisms and genes in the SML and SSW samples from the East China Sea and the Yellow Sea in summer. **(A)**, The abundance of *dddP* determined by qPCR. **(B)**, The abundance of *dmdA* determined by qPCR. **(C)**, The abundance of genera with representatives known to contain *dddP*. **(D)**, The abundance of genera with representatives known to contain *dmdA*. Three technical replicates are set for each sample. The SML and SSW samples are indicated with “m” or “s” in their sample names, respectively. YS_SML, the Yellow Sea SML samples; ECS_SML, the East China Sea SML samples; YS_SSW, the Yellow Sea SSW samples; ECS_SSW, the East China Sea SSW samples.

For DMSP catabolic genes in the eastern Chinese marginal sea, *dddP* and *dmdA* (C/2 and D/1) were more abundant in the SML (3.86 ± 4.05 × 10^7^ copies L^−1^ and 4.99 ± 7.69 × 10^8^ copies L^−1^) than those in the SSW samples (6.16 ± 8.75 × 10^6^ copies L^−1^ and 9.28 ± 9.33 × 10^7^ copies L^−1^; ~6.27-fold and 5.38-fold, *p* < 0.01, Wilcoxon signed-rank tests, Figures 6A,B; Supplementary Figures 7C,D; Supplementary Table 2). Both *dddP* and *dmdA* genes showed higher abundance in the FL fractions than in the PA fractions (Figures 6A,B), and *dmdA* was more abundant (~12.92-fold for the SML and ~15.06-fold for the SSW) than *dddP*, indicating that *dmdA*-mediated demethylation is the main pathway of DMSP catabolism in the eastern Chinese marginal sea. Additionally, *dmdA* D/1 subclade was far more abundant than C/2 subclade both in the SML and SSW (Figure 6B; Supplementary Figure 7D). The *dmdA* D/1 subclades (especially the FL fraction) was negatively correlated with longitude, DMS, and DMSP in the SML (*p* < 0.05, Spearman correlation tests, Supplementary Tables 7, 8).

Geographically, the abundance of *dmdA* (D/1 and C/2) in both SML and SSW of the ECS was higher (6.19 ± 8.74 × 10^8^ copies L^−1^ for SML and 9.64 ± 10.02 × 10^7^ copies L^−1^ for SSW, respectively) than that of the YS (1.69 ± 1.17 × 10^8^ copies L^−1^ for SML and 8.29 ± 8.36 × 10^7^ copies L^−1^ for SSW, respectively, Figure 6B; Supplementary Figure 7D). Similarly, the abundance of *dddP* in SML and SSW of the ECS was also both higher (4.29 ± 4.61 × 10^7^ copies L^−1^ for SML and 8.04 ± 9.60 × 10^6^ copies L^−1^ for SSW, respectively) than that of the YS (2.70 ± 1.83 × 10^7^ copies L^−1^ for SML and 9.93 ± 9.82 × 10^5^ copies L^−1^ for SSW, respectively, Figure 6A; Supplementary Figure 7C). DMSP metabolic genes (*dddP* and *dmdA*, especially in the FL fraction) in the SML of the ECS rather than the YS were negatively correlated with longitude (*p* < 0.05, Spearman correlation tests, Supplementary Tables 9–12). Compared with the spring ECS samples, the abundance of *dddP* and *dmdA* genes in summer SML was higher (~8.73-fold and ~38.56-fold, *p* < 0.05, Mann–Whitney tests, Supplementary Table 5).

### Abundance of potential DMSP producing and degrading genera in SML and SSW

16S rRNA gene abundance (qPCR) was used to evaluate the absolute abundance of DMSP producing and catabolic genera in SML and SSW samples of the eastern Chinese marginal sea (Dickson et al., 1980; Sunda et al., 2002; Curson et al., 2017; Williams et al., 2019). The DMSP biosynthetic genera *Ruegeria* (with *dsyB* and *mmtN*), *Alteromonas* (with *mmtN*) and *Croceicoccus* (with *mmtN*) were the most abundant in the eastern Chinese marginal sea, and their relative abundances in the SML were much higher (3.67%, 2.88%, and 0.19%) than that in the SSW (3.11%, 2.07%, and 0.14%, Figures 5C,D). However, the distribution of DMSP biosynthetic genera was significantly different in the ECS and YS. For the ECS samples, the DMSP biosynthetic genera that may contain *dsyB* included *Thalassobius*, *Oceanicola*, *Hoeflea* and *Albimonas*, as well as *Ruegeria*, *Roseovarius* and *Labrenzia* which may contain both *dsyB* and *mmtN* (Williams et al., 2019) were higher (~1.28–8.51 folds) in SML than SSW samples (*p* < 0.05, Wilcoxon rank-sum test, Figures 5C,D). *Thalassobaculum* containing *dsyB* (Williams et al., 2019) only presented in near shore SML sample (D3 and W1), but were not found in SSW. *Alteromonas*, *Croceicoccus* and *Novosphingobium*, containing *mmtN* (Williams et al., 2019), were ~4.46–7.32 fold higher in SML than SSW samples (*p* < 0.05, Wilcoxon rank-sum test, Figure 5D). The genus *Marinobacter*, which can produce DMSP but the synthesis gene is unknown, was also more abundant (~4.82-fold for ECS, *p* < 0.05, Wilcoxon rank-sum test) in SML than SSW samples. As for the samples from YS, *Ruegeria*, *Croceicoccus*, *Marinobacter*, and *Alteromonas* was also more abundant in SML than that of SSW, and *Thalassobaculum*, *Novosphingobium*, and *Streptomyces* only presented in SML samples. In contrast, *Thalassospira* and *Nisaea* only presented in SSW samples (Figures 5C,D). Most of the DMSP-synthesis bacteria (except that *Nisaea*, only presented in YS) were more abundant in the ECS than those in the YS (Figure 5). In contrast to the spring samples from ECS, the DMSP-synthesis bacteria were more diverse and more abundant in summer, and *Oceanicola*, *Thalassobius*, and *Thalassobaculum* only appeared in summer samples. The abundance of *Ruegeria* was higher in summer than in spring samples from the ECS (Figures 5C,D; Sun et al., 2020).

The relative abundance of DMSP consumers, e.g., *Roseobacter* clade bacteria (*Sulfitobacter*, *Paracoccus*, *Rugeria*, *Phaeobacter*, *Pseudophaeobacter*), SAR11 clade, SAR116 clade, were higher (range from 1.02 to 14.77 folds) in SML than in SSW in the eastern Chinese marginal sea (Figures 6C,D). The distribution of DMSP-catabolic bacteria differed between the ECS and the YS. *Loktanella*, *Thalassobius*, *Roseovarius* and *Labrenzia* were more abundant (~1.83–8.51-fold) in SML of the ECS than in SSW, which were inverse in the YS. In contrast, we found higher abundance of *Rubellimicrobium* in the YS SML (containing *dddP* genes, ~4.14-fold higher than in the SSW). The abundance of most DMSP-demethylation and cleavage genera in the ECS were much higher than that in the YS, such as *Roseobacter* clade bacteria, SAR11 clade and *Pseudophaeobacter* (Figures 6C,D). *Shimia* containing *dddP* and *dmdA* only presented in the ECS sample, but were not found in the YS. In both summer and spring ECS, some representative DMSP-degrading bacteria such as *Sulfitobacter*, *Paracoccus*, *Rugeria*, *Labrenzia*, *Shimia*, SAR11 and SAR116 were far more abundant in SML than in SSW samples (Sun et al., 2020), However, DMSP-degrading bacteria were more abundant during the summer than in the spring, and *Phaeobacter*, *Thalassobius*, and *Pseudophaeobacter* only appeared in summer but not in spring (Figures 6C,D; Sun et al., 2020).

## Discussion

As a ubiquitous sulfur-containing organic compound in the oceans, DMSP is of great significance in participating in the global sulfur cycle and regulating biogeochemical cycles in the oceans (Ksionzek et al., 2016). SML is the interface where exchanges occur between the ocean and atmosphere, and the existence of surface tension makes it physically stable, but it is also more susceptible to environmental and climate changes than SSW (Hardy, 1982). In turn, microorganisms and environmental factors in SML also affect the air-sea exchange process (Zäncker et al., 2018). Understanding microbial processes of the SML could make a vital contribution to mitigate these environmental changes (Engel et al., 2017). In this study, we focused on the spatiotemporal differences of DMSP metabolic bacteria in the SML of eastern Chinese marginal sea. Our results indicated that although there were obvious differences between regions and seasons, the activity of bacterial DMSP metabolism is consistently higher in the SML.

### Spatiotemporal distributions of DMS and DMSP in the SML and SSW samples

DMSP concentrations in SML were higher than that of SSW in both summer and spring, while no significant difference for DMS levels between SSW and SML was detected, irrespective of seasonality. The higher DMSP concentration could be related to the highly active DMSP biosynthesis in the SML. Although the DMSP cleavage pathway was also more active in the SML, the rapidly release of DMS from SML may lead to the observed similar DMS level in the SML and SSW. Seasonally, it is commonly reported the concentration of DMS or DMSP are higher in summer than in spring (Jian et al., 2019; Mao et al., 2021), which is consistent with our findings that the concentrations of DMS and total DMSP in SML of the ECS in summer were more abundant than those in spring (Supplementary Table 1; Sun et al., 2020). Additionally, the Chl *a* concentration of the ECS SML in summer was higher (~2.00-fold) than that in spring, reflecting the higher primary productivity and more active biological metabolism in summer, and this could be an explanation for the higher DMSP/DMS concentration in summer. However, there was no significant difference for Chl *a* level between SSW and SML (Supplementary Tables 3, 4), and no significant correlation was found between DMSP concentration and Chl *a* in both SML and in SSW. Combined with the study by Sun et al. (2020) in the SML of the ECS, it reinforces the idea that heterotrophic bacteria instead of eukaryotic algae, may have an important contribution to the higher DMSP concentration in SML.

### Spatiotemporal changes of bacteria in the SML and SSW samples

The total bacterial abundance of the SML samples in the eastern Chinese marginal sea in summer was significantly higher (~4.58-fold) than that of SSW samples (Supplementary Figure 2B), which was consistent with the results of the ECS (~7.50-fold) in spring (Sun et al., 2020). This is also consistent with Sieburth et al. (1976) who found a ~10^2^–10^4^-fold bacterial enrichment in SML from the North Atlantic compared with SSW samples. The higher bacterial abundance in SML may be due to its higher concentrations of nutrients and organic matter, higher temperature, as well as the physically stable environment under the action of surface tension (Kuznetsova et al., 2004; Zhang et al., 2006; Galachyants et al., 2018). The abundance of bacteria in the ECS was higher (~2.02-fold) than that in the YS. The higher ECS bacteria abundance was most likely the result of the regional hydrography including Taiwan Warm Current and Changjiang plume flowing northward near the Yangtze Estuary, the joint influence of the Yellow Sea cold water mass and the Yellow Sea warm current, as well as the discharge of Yangtze Diluted Water (Lee and Chao, 2003; Mi et al., 2012; Yeh et al., 2015; Yu et al., 2021). Bacteria were more abundant (~3.28-fold) in summer than those in spring from the ECS (Sun et al., 2020), and the SML bacteria decreased with the increase of offshore distance in summer. This is consistent with Zhao (2014) who found bacterial abundance in summer were higher than those in spring in both the SML and SSW samples from the North Yellow Sea, and the SML bacteria were more abundant in the nearshore area in summer. This may be due to generally higher levels of available nutrients in the nearshore area (Maki, 2003) and higher primary productivity in summer.

The relative abundance of *Gammaproteobacteria* in the eastern Chinese marginal sea was higher in the SML compared to the SSW, while there was no significant difference in *Alphaproteobacteria* between the two layers (Figure 3; Supplementary Figure 3A). On the contrary, Sun et al. (2020) found that *Alphaproteobacteria* were more abundant in the SSW of the ECS in spring. Many genera, *Pseudoalteromonas*, *Erythrobacter*, *Psychrobacter*, *Vibrio*, *Halomonas* and *Pseudomonas* were more abundant in the SML samples (Supplementary Figure 3B). There were seasonal and regional differences in bacterial community composition between SML and SSW samples. Most genera, such as *Erythrobacter*, *Ruegeria*, *Pseudoalteromonas*, *Alteromonas*, *Halomonas*, *Cobetia*, *Acinetobacter*, *Marinobacter* and *Vibrio* were significantly higher in ECS SML samples compared with SSW samples both in summer and spring (Supplementary Figure 6B; Sun et al., 2020). However, the diversity and richness of bacterial community structure in SML and SSW samples of ECS in summer were significantly higher than those in spring (Supplementary Figures 5C,D). The relative abundances of *Alphaproteobacteria*, *Flavobacteria*, *Cyanobacteria*, *Actinobacteria*, *Sphingobacteriia* and *Bacteroidia* in the ECS SML were higher compared to the YS SML, whereas the relative abundances of *Gammaproteobacteria* and *Bacilli* were opposite (Figure 3), which may be due to the variation in levels of available nutrients, pH, DO, DOC, Chl *a* and temperature in different marine areas (Supplementary Table 5).

### Active bacterial DMSP production in the ECS SML in summer

Correlated to the high DMSP concentrations, both the abundance of bacteria containing *dsyB* and *mmtN* (Figures 5A,B) and the abundance of DMSP-producing genera were higher in the SML samples (Figures 5C,D). Several DMSP biosynthesis genera, such as *Thalassobaculum*, were only found in the SML samples. At the gene level, *dsyB* and *mmtN* were more abundant in the SML in both regions and seasons, and were consistent with previous studies (Sun et al., 2020, 2021), the transamination pathway catalyzed by *dsyB* is the main DMSP production process in both SML and SSW. Furthermore, although *dsyB* and *mmtN* were more abundant in FL than in PA bacteria in spring ESC samples (Sun et al., 2020), no difference for *mmtN* was observed between the two lifestyles in summer samples (Figures 5A,B). However, considering the difference of abundant *dsyB* in FL and PA bacteria, FL bacteria could still be the main DMSP producers in the SML. Additionally, the major DMSP producing genera may be different between spring (*Alteromonas*) and summer (*Ruegeria*) of ESC (Sun et al., 2020).

Regionally, DMSP-production genes and the abundance and diversity of the corresponding DMSP-production bacteria were lower in the YS compared with the ECS (lower *dsyB* abundance, but no significant difference was found for *mmtN*). This may be due to the strong invasion of Kuroshio increases salinity in the summer ECS, phytoplankton and bacteria will produce more DMSP to balance intracellular osmotic pressure and thus the higher DMSP-synthesis bacteria and genes in the ECS (Sun et al., 2021).

### Seasonal and regional variations in bacterial DMSP catabolism in SML and SSW samples

The DMSP-catabolic genes (*dddP* and *dmdA*) and the corresponding bacteria (most of Roseobacter clade bacteria, SAR11 clade and SAR116 clade) were significantly more abundant in SML of the eastern Chinese marginal sea compared with SSW samples potentially indicating that bacterial DMSP catabolism more active in the SML. *dmdA* (C/2 and D/1 subclades) was higher than *dddP* in both summer and spring (Sun et al., 2020). This is consistent with Liu et al. (2018) who also found the genetic potential to cleave DMSP *via* the DddP DMSP lyase was far less prominent than that for DMSP demethylation in the ECS. It indicates that DMSP demethylation pathway may be more prominent than cleavage pathway. *dmdA* D/1 subclade in summer and *dmdA* C/2 subclade in spring had more potential to demethylate DMSP in the SML (Sun et al., 2020), indicating that the seasonal variation would affect the DMSP metabolism in gene subclades.

Consistent with the DMSP-synthesis gene (*dsyB*), DMSP-degradation genes (*dddP* and *dmdA*) in FL fraction were also more abundant than that in PA fraction. This is in agreement with Sun et al. (2020) who showed that the *dmdA* and *dddP* genes of FL bacteria was higher than PA bacteria in spring ECS. The *dmdA* D/1 subclades in the FL fraction was negatively correlated with DMSP in the SML of the eastern Chinese marginal sea (Supplementary Table 8). FL bacteria but not PA bacteria containing *dmdA* were significantly correlated with DMS and DMSP_t_ concentrations in ECS SML, and with DMSP_p_ concentration in ECS SSW (Supplementary Table 9). FL bacteria related to *dddP* and *dmdA*, but not PA bacteria, were correlated with DMSP_d_ concentration in both SML and SSW of the YS (Supplementary Table 12). Sun et al. (2020) also found the positive correlation between *dddP* and *dmdA* genes in FL bacteria and DMSP concentrations in SSW of the ECS in spring. These results indicate that FL bacteria may become the main DMSP consumers in the eastern Chinese marginal sea *via* the demethylation and cleavage pathways.

*dmdA* and *dddP* in SML and SSW samples from ESC was higher than that in the YS, which is consistent with the abundance of DMSP metabolic bacteria (*Ruegeria*, *Sulfitobacter*, *Shimia* and SAR11 clade; Figure 6). This result implied that the ECS may have a higher DMSP degradation potential compared with the YS. Furthermore, the significant negative correlation between the *dddP* and *dmdA* genes of FL bacteria and longitude in the ECS SML but not in the YS indicates that the DMSP-degradation capacity of the SML in the ECS may decrease with the increase of offshore distance. DMSP metabolic bacteria (*Phaeobacter*, *Thalassobius* and *Pseudophaeobacter*) and genes (*dmdA* and *dddP*) were higher in summer than spring in ECS (Sun et al., 2020). These results were also consistent with Kudo et al. (2018), who found *dddP* to be more abundant in summer compared to spring samples from Ofunato Bay, indicating that DMSP metabolic potential was more abundant in summer.

## Conclusion

The current study describes the spatiotemporal and seasonally differences of DMSP/DMS content, DMSP synthetic and catabolic bacteria and their functional genes in SML and SSW seawater samples of the eastern Chinese marginal sea. DMSP level, total bacteria, bacterial genera known to produce DMSP and their related DMSP synthesis genes, *dsyB* and *mmtN*, were more abundant in SML than in SSW for the eastern Chinese marginal sea compared with the SSW. Regarding DMSP catabolism, *dmdA* and *dddP* was also more abundant in SML than in SSW samples. *dsyB*, *dmdA* and *dddP* detected in SML and SSW of the East China Sea were significantly higher compared with the Yellow Sea, and the species and abundance of known DMSP synthesis and degradation genera were also more abundant in SML of the East China Sea. DMSP level and DMSP metabolic bacteria and genes were higher in SML of the ECS in summer than those in spring. Overall, this study revealed the distribution pattern of bacterial DMSP production and catabolic genes in the SML and SSW, and demonstrated that DMS/DMSP, DMSP-synthetic and catabolic bacteria as well as related genes exhibited spatiotemporal differences. These results elucidate that although the bacterial DMSP metabolism in the SML of the eastern Chinese marginal seas showed distinct spatiotemporal characteristics, the bacterial DMSP biosynthesis and catabolism are more active in the SML across different regions and seasons.

## Data availability statement

The datasets presented in this study can be found in online repositories. The names of the repository/repositories and accession number(s) can be found in the article/Supplementary material.

## Author contributions

X-HZ designed the experiments, analyzed the data, and wrote the manuscript. XL and YZ analyzed the data and wrote the manuscript. HS and ST collected samples and performed experiments. All authors contributed to the article and approved the submitted version.

## Funding

This work was supported by the National Natural Science Foundation of China (92251303 and 41730530), the Fundamental Research Funds for the Central Universities (202172002), and the Scientific and Technological Innovation Project of Laoshan Laboratory (2022QNLM030004-3, LSKJ202203201, and LSKJ202203206).

## Conflict of interest

The authors declare that the research was conducted in the absence of any commercial or financial relationships that could be construed as a potential conflict of interest.

## Publisher’s note

All claims expressed in this article are solely those of the authors and do not necessarily represent those of their affiliated organizations, or those of the publisher, the editors and the reviewers. Any product that may be evaluated in this article, or claim that may be made by its manufacturer, is not guaranteed or endorsed by the publisher.

## Footnotes

^1^http://www.arb-silva.de

^2^https://mothur.org/

^3^https://www.originlab.com
