# Supplementary material for: Highly active bacterial DMSP metabolism in the surface microlayer of the eastern China marginal seas
Source: Front Microbiol. 2023 Mar 23;14:1135083. doi: 10.3389/fmicb.2023.1135083 (PMC10076866; doi:10.3389/fmicb.2023.1135083)
Supplement: Supplementary file 1 [file Data_Sheet_1.pdf]

Supplementary materials

Highly active bacterial DMSP metabolism in the surface microlayer of the Eastern China Marginal Seas

Xiujie Liu[1†], Yunhui Zhang[3†], Hao Sun[1], Siyin Tan[1], and Xiao-Hua Zhang[1,2,3*]

[1]Frontiers Science Center for Deep Ocean Multispheres and Earth System, and College of Marine Life Sciences, Ocean University of China, Qingdao 266003, China

[2]Laboratory for Marine Ecology and Environmental Science, Laoshan Laboratory, Qingdao 266237, China

[3]Institute of Evolution & Marine Biodiversity, Ocean University of China, Qingdao 266003, China

*Correspondence:

Xiao-Hua Zhang, College of Marine Life Sciences, Ocean University of China, 5 Yushan Road, Qingdao 266003, PR China, Tel/Fax: +86-532-82032767, Email: xhzhang@ouc.edu.cn

† These authors contributed equally to this work.

Running title: Bacterial DMSP metabolism in surface microlayer

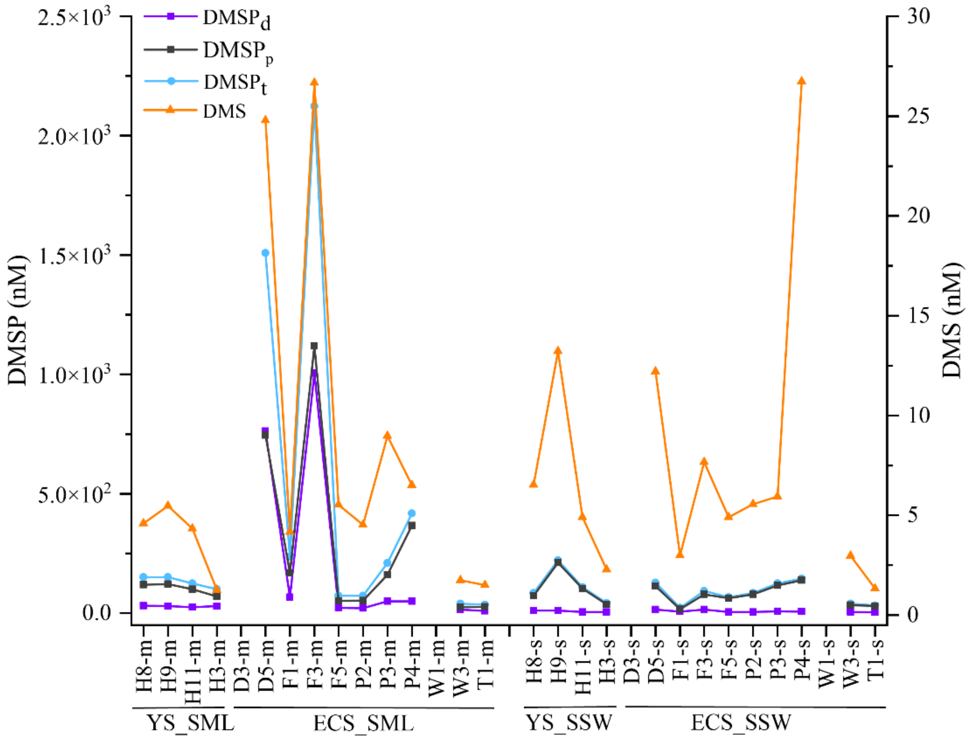

Supplementary Figure 1 The DMS and DMSP (total, particulate and dissolved) concentrations of SML and SSW in the eastern China marginal seas in summer (Ma and Yang, 2023). The SML and SSW samples are indicated with "m" or "s" in their sample names, respectively. YS_SML, the Yellow Sea SML samples; ECS_SML, the East China Sea SML samples; YS_SSW, the Yellow Sea SSW samples; ECS_SSW, the East China Sea SSW samples. The data of D3 and W1 sites were missing.

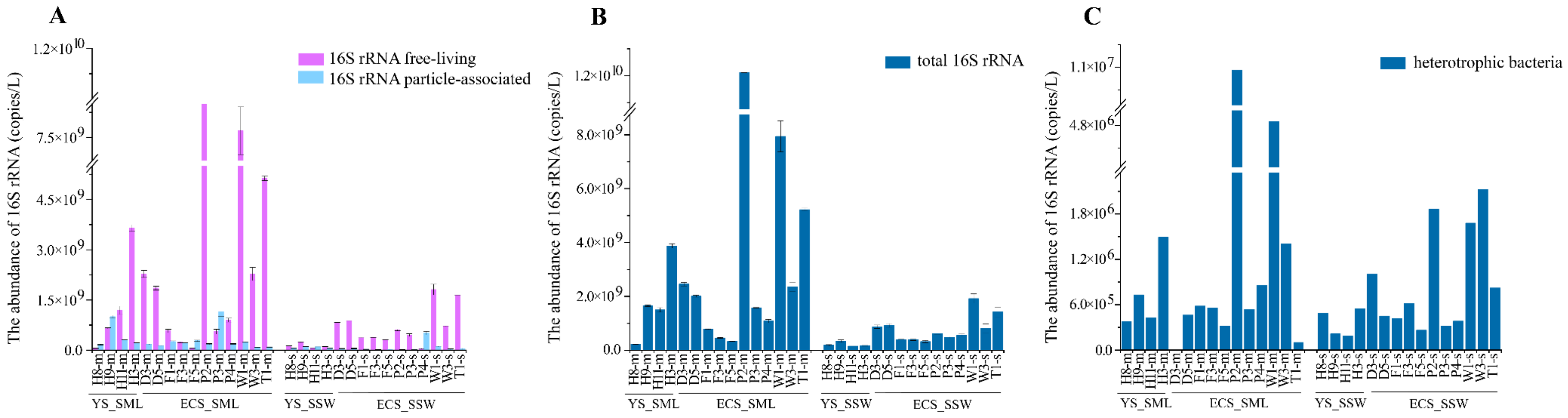

Supplementary Figure 2 The abundance of heterotrophic bacteria and 16S rRNA gene of the SML and SSW in the eastern China marginal seas determined in summer determined by qPCR. Three technical replicates are set for each sample. **(A)**, The abundance of 16S rRNA free-living and particle-associated gene. **(B)**, The abundance of 16S rRNA gene. **(C)**, The abundance of heterotrophic bacteria. The SML and SSW samples are indicated with "m" or "s" in their sample names, respectively. ECS_SML, the East China Sea SML samples; ECS_SSW, the East China Sea SSW samples; YS_SML, the Yellow Sea SML samples; YS_SSW, the Yellow Sea SSW samples.

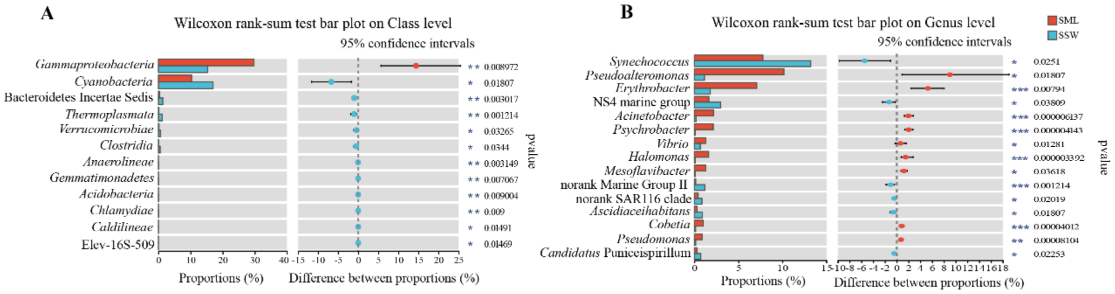

Supplementary Figure 3 The differences of bacterial community structure in the SML and SSW of the eastern China marginal seas in summer. **(A)**, Wilcoxon rank-sum test bar plot on class level of the eastern China marginal seas in summer; **(B)**, Wilcoxon rank-sum test bar plot on genus level of the eastern China marginal seas in summer. *, $p < 0.05$; **, $p < 0.01$; ***, $p < 0.001$.

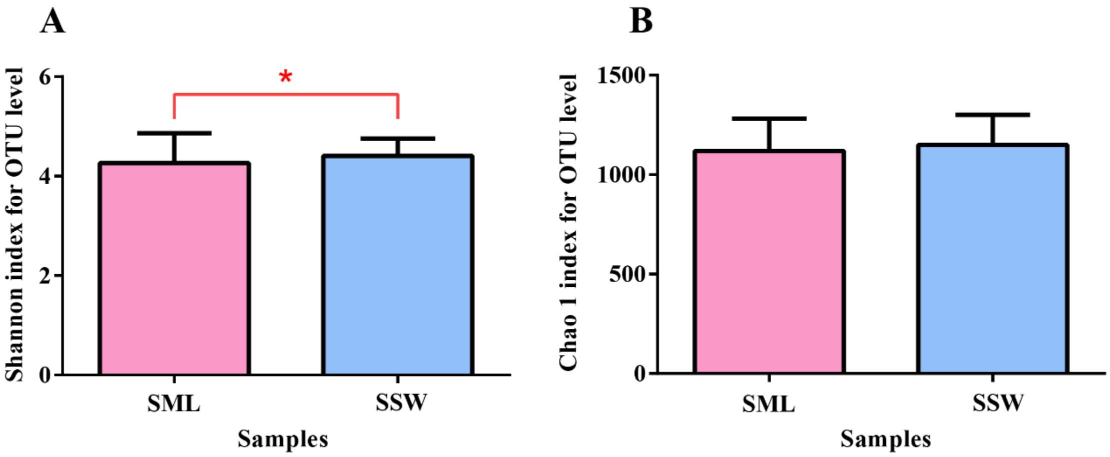

Supplementary Figure 4 The difference of Shannon and Chao 1 index of the eastern China marginal seas between SML and SSW in summer. **(A)**, the difference of Shannon index of the eastern China marginal seas between SML and SSW samples in summer. **(B)**, the difference of Chao 1 index of the eastern China marginal seas between SML and SSW samples in summer. *, $p < 0.05$; **, $p < 0.01$; ***, $p < 0.001$.

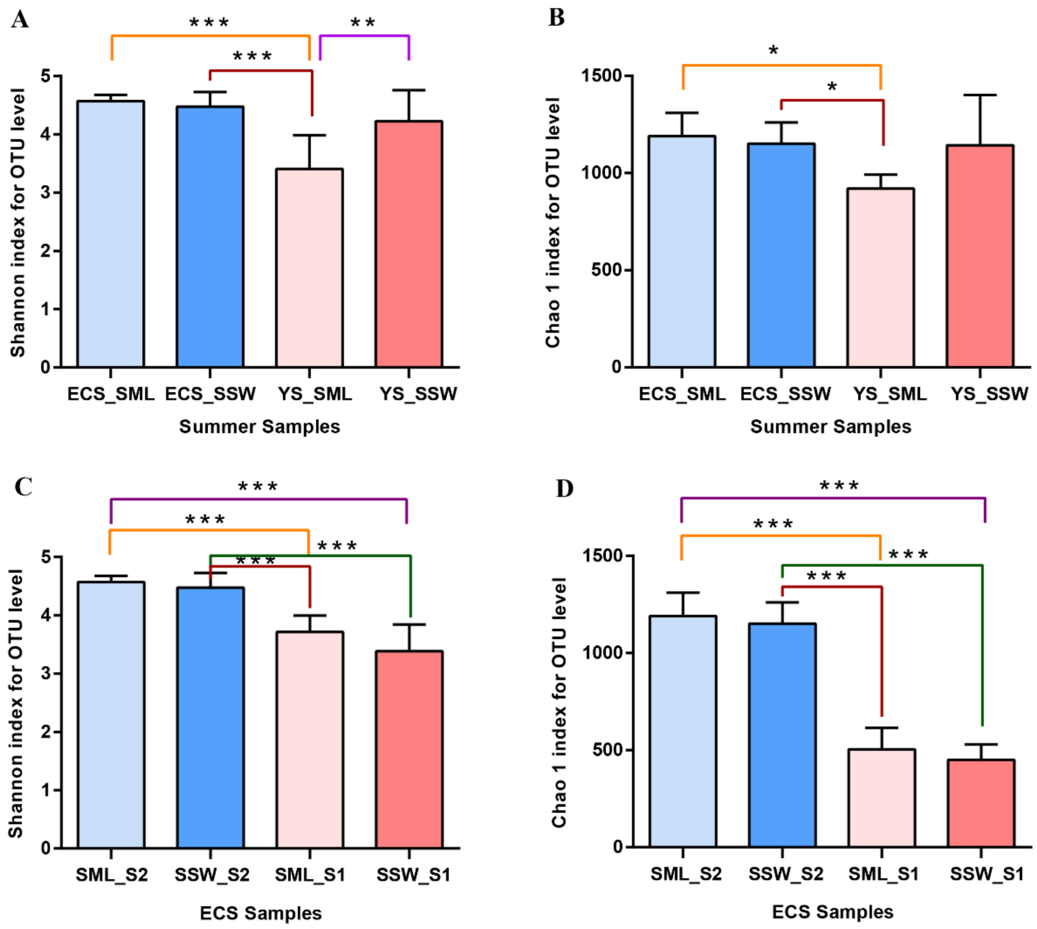

Supplementary Figure 5 The difference of Shannon and Chao 1 index of SML and SSW between ECS and YS and between summer and spring. **(A)**, the difference of Shannon index of SML and SSW between ECS and YS in summer samples. **(B)**, the difference of Chao 1 index of SML and SSW between ECS and YS in summer samples. **(C)**, the difference of Shannon index of SML and SSW between summer and spring in ECS samples. **(D)**, the difference of Chao 1 index of SML and SSW between summer and spring in ECS samples. ECS_SML, the East China Sea SML samples; ECS_SSW, the East China Sea SSW samples; YS_SML, the Yellow Sea SML samples; YS_SSW, the Yellow Sea SSW samples; SML_S1, the SML samples in spring; SML_S2, the SML samples in summer; SSW_S1, the SSW samples in spring; SSW_S2, the SSW samples in summer. *, $p < 0.05$; **, $p < 0.01$; ***, $p < 0.001$.

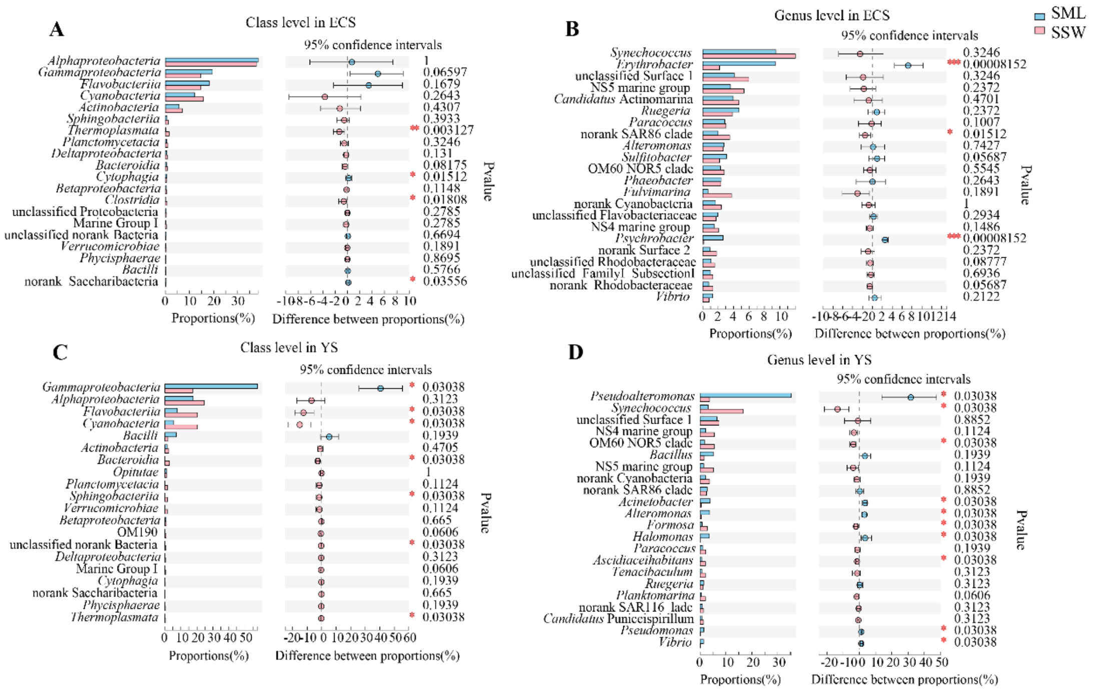

Supplementary Figure 6 The differences of bacterial community structure in the SML and SSW of the East China Sea and the Yellow Sea in summer. **(A)**, Wilcoxon rank-sum test bar plot on class level of the East China Sea in summer; **(B),** Wilcoxon rank-sum test bar plot on genus level of the East China Sea in summer; **(C)**, Wilcoxon rank-sum test bar plot on class level of the Yellow Sea in summer; **(D),** Wilcoxon rank-sum test bar plot on genus level of the Yellow Sea in summer. *, $p < 0.05$; **, $p < 0.01$; ***, $p < 0.001$.

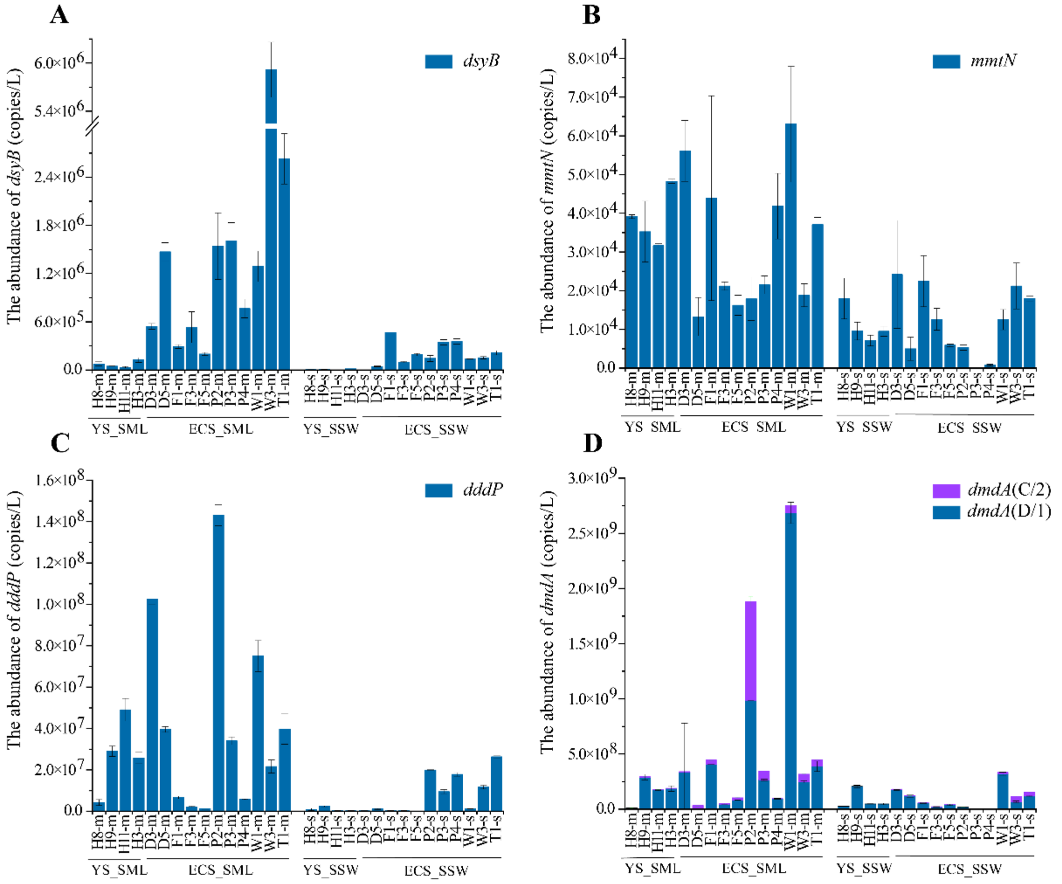

Supplementary Figure 7 The abundance of DMSP-producing and catabolic genes in SML and SSW samples from the East China Sea and the Yellow Sea in summer determined by qPCR. Three technical replicates are set for each sample. **(A)**, The abundance of *dsyB* gene. **(B)**, The abundance of *mmtN* gene. **(C)**, The abundance of *dddP* gene. **(D)**, The abundance of *dmdA* gene. The SML and SSW samples are indicated with "m" or "s" in their sample names, respectively. ECS_SML, the East China Sea SML samples; ECS_SSW, the East China Sea SSW samples; YS_SML, the Yellow Sea SML samples; YS_SSW, the Yellow Sea SSW samples.

Supplementary Tables

Supplementary Table 1 The environmental parameters and microplankton of the SML and SSW samples in the East China Sea and the Yellow Sea.

Station	sampling date	Longitude (°E)	Latitude (°N)	Depth (m)	Salinity (PSU)	Temp (°C)	pH	DO (mg/l)	Chl *a* (μg/l)	SiO_3^{2-} (μM)	NO_2^- (μM)	NH_4^+ (μM)	PO_4^{3-} (μM)	NO_3^- (μM)	DOC (μM)	DMS (nM)	$DMSP_t$ (nM)	$DMSP_d$ (nM)	$DMSP_p$ (nM)	SYN (cells/mL)	PEUK (cells/mL)	HB (cells/mL)
H8-s	2018.6.28	121.42	38.46	3.20	31.79	19.44	8.06	8.94	1.30	N.D.	0.01	0.11	0.04	N.D.	146.33	6.52	10.49	73.07	83.56	1470	262	484647
H9-s	2018.6.28	122.05	38.75	3.00	32.13	20.68	8.13	8.23	0.48	N.D.	0.03	N.D.	0.02	N.D.	87.00	13.22	10.90	210.61	221.51	1670	283	213753
H11-s	2018.6.29	123.80	38.75	2.60	32.06	20.24	8.14	8.05	0.45	N.D.	0.04	N.D.	0.02	N.D.	138.75	4.90	4.77	103.52	108.29	3953	223	187404
H3-s	2018.6.29	123.97	37.00	2.80	31.75	21.84	8.12	8.24	0.18	N.D.	0.06	0.06	0.03	N.D.	128.25	2.28	5.26	36.71	41.97	979	30	544647
D3-s	2018.7.4	123.00	31.90	3.50	25.18	24.23	8.32	8.74	2.19	22.78	0.86	0.92	0.15	18.14	88.00	N.D.	N.D.	N.D.	N.D.	52120	5881	1002052
D5-s	2018.7.16	123.96	32.45	4.30	30.54	27.18	8.30	7.22	0.57	3.15	0.28	0.19	0.07	2.09	93.67	12.21	14.80	112.82	127.62	4397	31	449194
F1-s	2018.7.5	122.97	31.33	2.80	29.36	24.79	8.10	6.09	3.79	4.03	4.03	0.09	9.97	0.84	95.83	2.98	5.79	16.72	22.51	1008	114	418873
F3-s	2018.7.5	124.35	31.33	2.60	31.68	26.26	8.27	7.49	1.04	N.D.	N.D.	0.08	0.62	0.05	102.08	7.66	14.54	78.20	92.74	3827	18	613420
F5-s	2018.7.6	126.20	31.33	4.10	32.00	24.23	8.19	7.42	0.47	6.68	6.68	0.02	N.D.	N.D.	107.33	4.91	4.33	62.99	67.32	4724	229	263510
P2-s	2018.7.16	123.13	30.92	2.60	31.74	27.02	8.29	7.84	2.47	5.49	0.03	0.18	0.05	0.25	87.33	5.56	4.83	79.08	83.92	48048	6	1862496
P3-s	2018.7.16	123.60	30.40	3.30	31.74	27.76	8.29	6.95	1.35	3.22	0.04	0.08	0.05	N.D.	103.08	5.95	7.80	116.33	124.13	8104	10	313776
P4-s	2018.7.16	124.30	30.00	5.00	33.08	28.10	8.31	7.91	1.21	1.14	0.05	0.07	0.04	N.D.	76.70	26.73	6.39	138.32	144.72	10036	6	383376
W1-s	2018.7.15	122.00	28.60	2.50	30.38	27.35	8.16	6.57	3.75	12.35	1.06	1.46	0.51	8.99	102.83	N.D.	N.D.	N.D.	N.D.	43744	4204	1679550
W3-s	2018.7.14	122.85	27.90	4.40	34.11	27.39	8.17	7.01	0.28	1.64	N.D.	0.00	0.04	N.D.	80.24	2.97	4.86	34.15	39.01	683	58	2122500
T1-s	2018.7.13	120.48	26.87	3.00	34.02	26.28	8.15	6.47	0.96	3.42	0.40	0.05	0.21	0.48	88.08	1.33	2.82	29.23	32.05	11338	257	821478
H8-m	2018.6.28	121.42	38.46	0	N.D.	N.D.	N.D.	N.D.	0.51	N.D.	N.D.	N.D.	N.D.	N.D.	187.17	4.58	31.25	119.32	150.57	1443	23	376800
H9-m	2018.6.28	122.05	38.75	0	N.D.	N.D.	N.D.	N.D.	0.46	N.D.	N.D.	N.D.	N.D.	N.D.	234.67	5.47	29.42	121.55	150.97	764	85	729294
H11-m	2018.6.29	123.80	38.75	0	N.D.	N.D.	N.D.	N.D.	0.73	N.D.	N.D.	N.D.	N.D.	N.D.	172.42	4.34	25.34	99.40	124.74	3172	53	427149
H3-m	2018.6.29	123.97	37.00	0	N.D.	N.D.	N.D.	N.D.	0.29	N.D.	N.D.	N.D.	N.D.	N.D.	188.75	1.26	29.24	70.40	99.64	1485	11	1491472
D3-m	2018.7.4	123.00	31.90	0	N.D.	N.D.	N.D.	N.D.	4.01	N.D.	N.D.	N.D.	N.D.	N.D.	N.D.	N.D.	N.D.	N.D.	N.D.	N.D.	N.D.	N.D.
D5-m	2018.7.16	123.96	32.45	0	N.D.	N.D.	N.D.	N.D.	1.83	N.D.	N.D.	N.D.	N.D.	N.D.	137.42	24.79	762.59	745.33	1507.92	5481	84	463164
F1-m	2018.7.5	122.97	31.33	0	N.D.	N.D.	N.D.	N.D.	3.59	N.D.	N.D.	N.D.	N.D.	N.D.	197.58	4.16	67.32	168.10	235.42	2204	102	581388
F3-m	2018.7.5	124.35	31.33	0	N.D.	N.D.	N.D.	N.D.	1.63	N.D.	N.D.	N.D.	N.D.	N.D.	161.42	26.67	1005.17	1118.78	2123.95	4761	33	556310
F5-m	2018.7.6	126.20	31.33	0	N.D.	N.D.	N.D.	N.D.	0.50	N.D.	N.D.	N.D.	N.D.	N.D.	128.33	5.52	21.74	50.87	72.62	3441	27	316898
P2-m	2018.7.16	123.13	30.92	0	N.D.	N.D.	N.D.	N.D.	2.72	N.D.	N.D.	N.D.	N.D.	N.D.	114.08	4.53	20.45	52.09	72.54	44994	2	11360400
P3-m	2018.7.16	123.60	30.40	0	N.D.	N.D.	N.D.	N.D.	1.76	N.D.	N.D.	N.D.	N.D.	N.D.	129.08	8.98	49.29	160.09	209.38	10148	4	538128
P4-m	2018.7.16	124.30	30.00	0	N.D.	N.D.	N.D.	N.D.	2.31	N.D.	N.D.	N.D.	N.D.	N.D.	122.75	6.51	49.62	367.66	417.28	20492	44	854160
W1-m	2018.7.15	122.00	28.60	0	N.D.	N.D.	N.D.	N.D.	2.67	N.D.	N.D.	N.D.	N.D.	N.D.	117.58	N.D.	N.D.	N.D.	N.D.	40588	996	4848150
W3-m	2018.7.14	122.85	27.90	0	N.D.	N.D.	N.D.	N.D.	0.25	N.D.	N.D.	N.D.	N.D.	N.D.	85.67	1.75	14.32	25.73	40.05	654	94	1406350
T1-m	2018.7.13	120.48	26.87	0	N.D.	N.D.	N.D.	N.D.	0.81	N.D.	N.D.	N.D.	N.D.	N.D.	109.50	1.50	8.76	26.26	35.03	8526	75	101165

[a] "m" of the sample name means the SML sample, and "s" means the SSW sample, N.D. represents not done.

[b] DMS and DMSP data were provided by Ma and Yang (2023).

Supplementary Table 2 The *p* values of Wilcoxon signed-rank Tests for the difference of genes abundance and environmental factors between SML and SSW water samples of the entire eastern China marginal seas.

Genes and environmental factors	Bacteria	*dsyB*	*mmtN*	*dddP*	*dmdA* C/2	*dmdA* D/1	DOC	$DMSP_d$	$DMSP_t$
Total	**0.001**	**0.002**	**0.001**	**0.002**	**0.001**	**0.003**			
Particles	**0.008**		**0.002**	0.017	**0.001**		**0.001**	**0.001**	0.019
Free-living	**0.004**	**0.001**	**0.005**	**0.001**	**0.003**	**0.004**			

[a] Only significant differences ($p < 0.05$) were shown in table. Bold, $p < 0.01$; regular, $p < 0.05$.

Supplementary Table 3 The *p* values of Wilcoxon signed-rank Tests for the difference of genes abundance and environmental factors between SML and SSW water samples of the East China Sea.

Genes and environmental factors	Bacteria	*dsyB*	*mmtN*	*dddP*	*dmdA* C/2	*dmdA* D/1	DOC	$DMSP_d$	$DMSP_t$
Total	**0.003**	**0.006**	**0.004**	0.013	**0.003**	**0.010**			
Particles	0.041		**0.007**		**0.003**		**0.005**	**0.008**	0.028
Free-living	0.016	**0.003**	0.022	**0.004**	**0.010**	**0.010**			

[a] Only significant differences ($p < 0.05$) were shown in table. Bold, $p < 0.01$; regular, $p < 0.05$.

Supplementary Table 4 The *p* values of Wilcoxon signed-rank Tests for the difference of genes abundance and environmental factors between SML and SSW water samples of the Yellow Sea.

Genes and environmental factors	Bacteria	*dsyB*	*mmtN*	*dddP*	*dmdA* C/2	*dmdA* D/1	DOC	$DMSP_d$
Total	0.043	0.021	0.021	0.021	0.043			
Particles		0.021	0.034	0.021			0.021	0.021
Free-living		0.021	0.021	0.043				

[a] Only significant differences ($p < 0.05$) were shown in table. Bold, $p < 0.01$; regular, $p < 0.05$.

Supplementary Table 5 The p value of Mann-Whitney Tests for the differences of environmental parameters and genes abundance between seasons and between regions in SML and SSW.

Environmental parameters		Bacteria	*dsyB*	*mmtN*	*dddP*	*dmdA* C/2	*dmdA* D/1	Temp	pH	DO	DOC	Chl *a*	PO_4^{3-}	$DMSP_d$	SYN
Seasons (Spring and Summer)	SML		**0.017**	**0.021**	**0.017**	**0.003**	**0.003**				**0.002**			0.023	
	SSW	**0.001**	**0.026**	**0.048**			**0.006**	**0.001**		**0.007**	0.012				
Regions (ECS and YS)	SML		**0.004**			**0.013**					0.014	0.040			0.036
	SSW	**0.006**	**0.019**			**0.050**		**0.001**	**0.010**	**0.010**			**0.002**		

[a] Only significant differences ($p < 0.05$) were shown in table. Bold, $p < 0.01$; regular, $p < 0.05$.

Supplementary Table 6 The original sequence numbers, the OTU numbers after subsampling and the alpha diversity of the SML and SSW samples in the East China Sea and the Yellow Sea in summer.

Samples	Sequence numbers	OTU	Shannon	Chao 1	coverage
H8-m	94386	789	4.13	976.33	99.73%
H9-m	107303	664	3.61	957.78	99.66%
H11-m	68251	623	2.98	815.30	99.73%
H3-m	109110	653	2.91	930.57	99.67%
D3-m	136126	879	4.63	1076.64	99.66%
D5-m	68671	982	4.67	1258.25	99.65%
F1-m	136920	906	4.59	1270.00	99.58%
F3-m	76645	1006	4.77	1275.01	99.59%
F5-m	96996	930	4.49	1107.49	99.70%
P2-m	67857	785	4.46	964.16	99.72%
P3-m	135751	841	4.45	1100.10	99.65%
P4-m	83829	947	4.46	1136.73	99.68%
W1-m	95686	1027	4.48	1296.22	99.60%
W3-m	92609	1050	4.60	1269.43	99.63%
T1-m	79908	1152	4.67	1348.12	99.64%
H8-s	136897	1235	4.59	1448.33	99.61%
H9-s	90034	718	3.76	903.74	99.70%
H11-s	109480	803	3.78	955.48	99.71%
H3-s	81577	1087	4.78	1265.67	99.68%
D3-s	70794	992	4.73	1161.84	99.70%
D5-s	145518	860	4.22	1163.84	99.59%
F1-s	98164	1095	4.78	1315.28	99.59%
F3-s	101229	836	4.24	1115.35	99.63%
F5-s	91396	888	4.75	1034.06	99.76%
P2-s	72440	833	4.51	969.07	99.76%
P3-s	95133	990	4.47	1181.88	99.66%
P4-s	69048	959	4.45	1105.45	99.73%
W1-s	103384	791	3.98	1075.52	99.66%
W3-s	66827	1035	4.67	1218.99	99.70%
T1-s	89941	1110	4.43	1328.34	99.61%

[a] "m" of the sample name means the SML sample, and "s" means the SSW sample.

Supplementary Table 7 The correlations between total gene abundances and environmental factors of the entire eastern China marginal seas.

Environmental	parameters	Longitude	Latitude	Temperature	PO_4^{3-}	NO_2^-	NO_3^-	DMSPp	DMSPt	DO	DOC	SYN	PEUK
SML	*dsyB*		**-0.854**								**-0.811**		
	mmtN												
	dddP												
	C/2		**-0.646**								-0.591		
	D/1	-0.532											
	Bacteria												
SSW	*dsyB*		**-0.743**	**0.689**						**-0.754**			
	mmtN	-0.550						**-0.742**	**-0.703**				0.611
	dddP	-0.582		0.579							-0.575		
	C/2					0.665		-					
	D/1						**0.893**						**0.756**
	Bacteria		**-0.711**	**-0.671**	0.591					-0.532	-0.564	0.564	

[a] Only significant correlations were shown in table. Red, positive; blue, negative. Bold, $p < 0.01$; regular, $p < 0.05$.

Supplementary Table 8 The correlations between gene abundances and environmental factors in particle-associated and free-living bacteria of the eastern China marginal seas.

Environmental Parameters		SML								SSW											
		Latitude	DOC	Longitude	DMS	SYN	DMSPd	DMSPp	DMSPt	Longitude	Latitude	Temperature	DO	pH	NO_3^-	NO_2^-	DMS	DMSPp	DMSPt	PEUK	PO_4^{3-}
dsyB	Particles	**-0.796**	**-0.692**			0.622						0.586	-0.579							-0.565	
	Free-living	**-0.821**	**-0.749**								**-0.825**	**0.825**	**-0.721**								
mmtN	Particles																	-0.664			
	Free-living									**-0.650**				**-0.700**			-0.604	**-0.753**	**-0.736**	0.590	
dddP	Particles	0.521																			
	Free-living									-0.625	-0.525										
dmdA (C/2)	Particles																				
	Free-living		-0.613													0.626				0.518	
dmdA (D/1)	Particles																				
	Free-living			-0.529	**-0.703**		-0.593	-0.555	-0.604	-0.575										0.715	
Bacteria	Particles														0.821						
	Free-living										-0.543		-0.557								0.600

[a] Only significant correlations were shown in table. Red, positive; blue, negative. Bold, $p < 0.01$; regular, $p < 0.05$.

Supplementary Table 9 The correlations between gene abundances and environmental factors in particle-associated and free-living bacteria of the East China Sea.

Environmental		SML						SSW											
Parameters		Longitude	DOC	DMS	DMSPd	DMSPt	SYN	Longitude	Latitude	Temperature	DOC	pH	NO_3^-	NO_2^-	DMS	DMSPp	DMSPt	PEUK	HB
dsyB	Particles				-0.683	-0.733		0.609											-0.664
	Free-living								**-0.800**	**0.736**									
mmtN	Particles																	0.669	
	Free-living							-0.655				**-0.745**		0.717	**-0.800**	**-0.967**	**-0.917**	0.683	
dddP	Particles						0.697		-0.727	0.618	-0.609								
	Free-living	-0.783						-0.636											
dmdA	Particles																		
(C/2)	Free-living							-0.627							-0.733	-0.733		**0.793**	
dmdA	Particles																		
(D/1)	Free-living	**-0.773**		-0.783		-0.717		-0.709								-0.667		**0.843**	
Bacteria	Particles												0.821						
	Free-living	-0.718	-0.673					-0.700			0.636								

[a] Only significant correlations were shown in table. Red, positive; blue, negative. Bold, $p < 0.01$; regular, $p < 0.05$.

Supplementary Table 10 The correlations between total gene abundances and environmental factors of the East China Sea.

Environmental	parameters	Longitude	Latitude	Temperature	NO_2^-	NO_3^-	DMS	DMSPp	DMSPt	DOC	Salinity	PEUK	HB
SML	*dsyB*		-0.627							-0.685			
	mmtN												
	dddP	-0.609											
	C/2												
	D/1	**-0.736**					-0.683						
	Bacteria	-0.709								-0.685			
SSW	*dsyB*												
	mmtN			-0.645			-0.750	**-0.950**	**-0.883**			0.692	
	dddP		**-0.755**	0.655	-0.717						0.627		
	C/2	-0.609					-0.683	-0.717				**0.779**	
	D/1					**0.893**						**0.834**	
	Bacteria	-0.718											0.609

[a] Only significant correlations were shown in table. Red, positive; blue, negative. Bold, $p < 0.01$; regular, $p < 0.05$.

Supplementary Table 11 The correlations between total gene abundances and environmental factors of the Yellow Sea.

Environmental	parameters	Latitude	DOC	DMS	DMSPd	PEUK	HB
SML	*dsyB*	**-1.000**					
	mmtN	**-1.000**					
	dddP						
	C/2						
	D/1						
	Bacteria						**1.000**
SSW	*dsyB*						
	mmtN						
	dddP			**1.000**		**1.000**	
	C/2				**1.000**		
	D/1		**-1.000**				
	Bacteria				**1.000**		

[a] Only significant correlations were shown in table. Red, positive; blue, negative. Bold, $p < 0.01$; regular, $p < 0.05$.

Supplementary Table 12 The correlations between gene abundances and environmental factors in particle-associated and free-living bacteria of the Yellow Sea.

Environmental parameters		SML							SSW											
		Longitude	Chla	DMS	DMSPd	DMSPp	DMSPt	SYN	Longitude	Salinity	Temperature	pH	DO	DOC	PO_4^{3-}	NO_2^-	Chl a	DMSPp	DMSPd	DMSPt
dsyB	Particles																		**1.000**	
	Free-living		**-1.000**																	
mmtN	Particles		**-1.000**							**-1.000**	**-1.000**			**1.000**				**-1.000**		**-1.000**
	Free-living	**1.000**										**-1.000**	**1.000**		**1.000**					
dddP	Particles			**1.000**		**1.000**	**1.000**					**1.000**	**-1.000**		**-1.000**					
	Free-living				**-1.000**														**1.000**	
dmdA (C/2)	Particles			**1.000**		**1.000**	**1.000**		**1.000**							**1.000**	**-1.000**			
	Free-living																		**1.000**	
dmdA (D/1)	Particles							**-1.000**				**1.000**	**-1.000**		**-1.000**					
	Free-living																			
Bacteria	Particles																			
	Free-living	**1.000**																	**1.000**	

[a] Only significant correlations were shown in table. Red, positive; blue, negative. Bold, $p < 0.01$; regular, $p < 0.05$.

References

Ma, Q. Y., and Yang, G. P. (2023) Roles of phytoplankton, microzooplankton, and bacteria in DMSP and DMS transformation processes in the East China Continental Sea. *Prog. Oceanog.* 213. doi: 10.1016/j.pocean.2023.103003
